# Supplementary material for: The dynamics and functional impact of tRNA repertoires during early embryogenesis in zebrafish
Source: EMBO J. 2024 Oct 14;43(22):19. doi: 10.1038/s44318-024-00265-4 (PMC11574265; doi:10.1038/s44318-024-00265-4)
Supplement: Supplementary file 1 — Appendix [file 44318_2024_265_MOESM1_ESM.pdf]

Appendix for

The dynamics and functional impact of tRNA repertoires during early embryogenesis in zebrafish

Madalena M. Reimão-Pinto[1,#], Andrew Behrens[2,*], Sergio Forcelloni[2,*], Klemens Fröhlich[1], Selay Kaya[2] and Danny D. Nedialkova[2,3,#]

[1]Biozentrum, University of Basel, 4054 Basel, Switzerland

[2]Mechanisms of Protein Biogenesis Laboratory, Max Planck Institute of Biochemistry, 82152 Martinsried, Germany

[3]Technical University of Munich, TUM School of Natural Sciences, Department of Bioscience, 85748 Garching, Germany

[*]Contributed equally

[#]Correspondence: madalena.pinto@unibas.ch, nedialkova@biochem.mpg.de

Table of Contents

Page 2: Appendix Figure S1

Appendix Figure S1

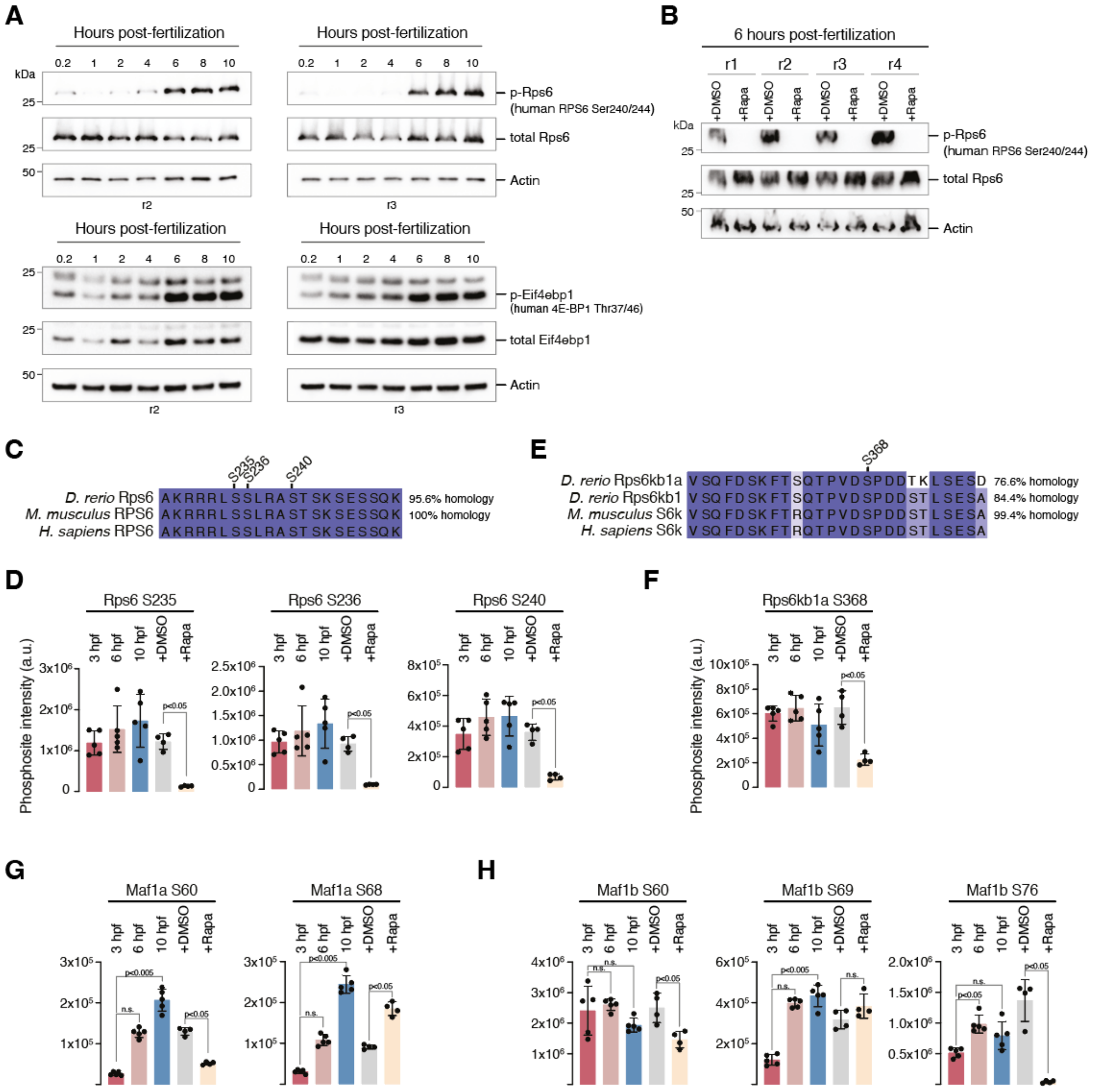

Appendix Figure S1. Analysis of TORC1-dependent phosphorylation during early zebrafish embryogenesis. (A) Replicate western blots of zebrafish embryo lysates during the maternal-to-zygotic transition (0.2 to 10 hpf). Protein molecular weight markers are specified on the left and antibodies are indicated on the right. Actin was used as a loading control. **(B)** Western blot of vehicle control (DMSO, 1%) and rapamycin-treated (Rapa, 1 μM) embryos at the shield stage (6 hpf) of the four independent replicates (r1 to r4) used for mass spectrometry analysis. Rps6 phosphorylation loss upon rapamycin treatment indicates TOR inhibition. Actin was used as loading control. **(C)** Pairwise alignment of human, mouse and zebrafish RPS6 protein sequences colored using the default Percentage Identity color scheme of Jalview. **(D)** Determination of phosphorylation levels at known conserved TORC1-dependent phosphosites by targeted mass spectrometry (see *Methods*). Bar plots indicating statistically significant differences between vehicle control (DMSO) and TORC1 inhibition (rapamycin) conditions at the shield stage (6 hpf) (Mann-Whitney U test, n = 4). Bars represent mean ± SD of untreated samples (n = 5) at 3, 6 and 10 hpf or of vehicle (DMSO) and rapamycin-treated (Rapa) samples (n = 4) at 6 hpf. **(E)** Pairwise alignment of human, mouse and zebrafish S6K protein sequences colored using the default Percentage Identity color scheme of Jalview. **(F)** as in D. **(G)-(H)** Determination of phosphorylation levels at conserved phosphosites in Maf1a or **(H)** Maf1b by targeted mass spectrometry (see *Methods*). These sites correspond to sites in the human MAF1 protein that were shown to be targeted by TORC1 (Michels *et al*, 2010; Shor *et al*, 2010). Bar plots indicating statistically significant differences between developmental time-points (3, 6 and 10 hpf; repeated measures ANOVA, n = 5) or between vehicle control (DMSO) and TORC1 inhibition (rapamycin) conditions at 6 hpf (Mann-Whitney U test, n = 4). Bars represent mean ± SD of untreated samples (n = 5) at 3, 6 and 10 hpf or of vehicle (DMSO) and rapamycin-treated (Rapa) samples (n = 4) at 6 hpf.
