# Supplementary figures and images for: The dynamics and functional impact of tRNA repertoires during early embryogenesis in zebrafish
Source: EMBO J. 2024 Oct 14;43(22):19. doi: 10.1038/s44318-024-00265-4 (PMC11574265; doi:10.1038/s44318-024-00265-4)

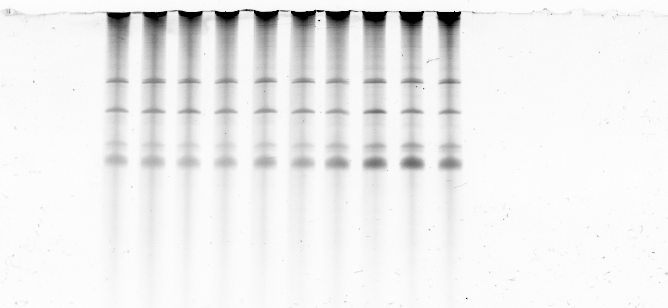

Supplement: Supplementary file 8 — Source data Fig. 2 [file 44318_2024_265_MOESM8_ESM.zip › SD_Figure2/Source_data_Figure2E/SYBR_Gold_Ala_AGC_3.tiff]

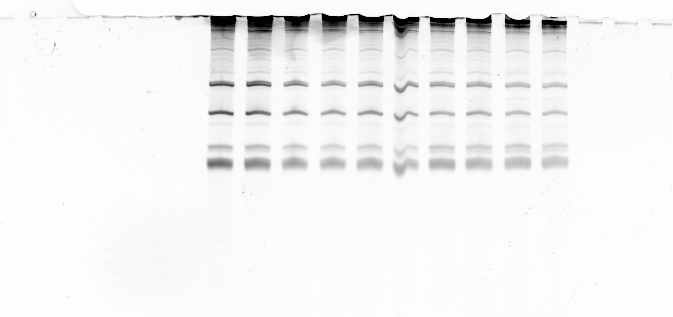

Supplement: Supplementary file 8 — Source data Fig. 2 [file 44318_2024_265_MOESM8_ESM.zip › SD_Figure2/Source_data_Figure2E/SYBR_Gold_Thr_UGU_8.tiff]

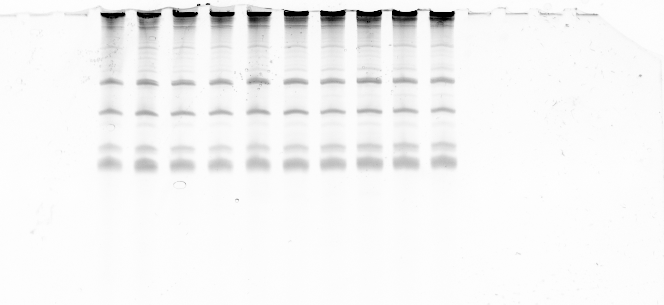

Supplement: Supplementary file 8 — Source data Fig. 2 [file 44318_2024_265_MOESM8_ESM.zip › SD_Figure2/Source_data_Figure2E/SYBR_Gold_Ser_UGA_19.tiff]

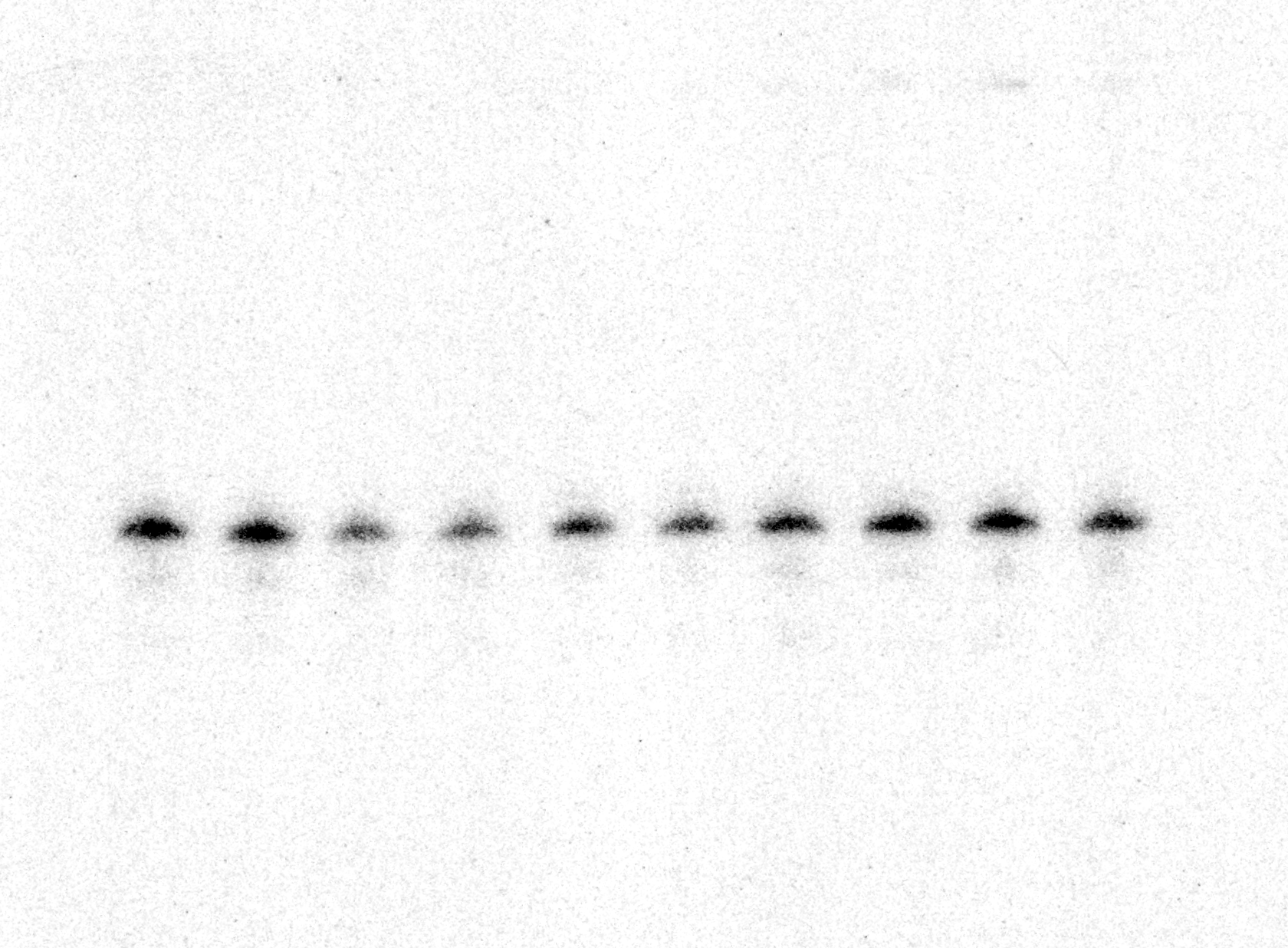

Supplement: Supplementary file 8 — Source data Fig. 2 [file 44318_2024_265_MOESM8_ESM.zip › SD_Figure2/Source_data_Figure2E/northern_Ala_AGC_3.tiff]

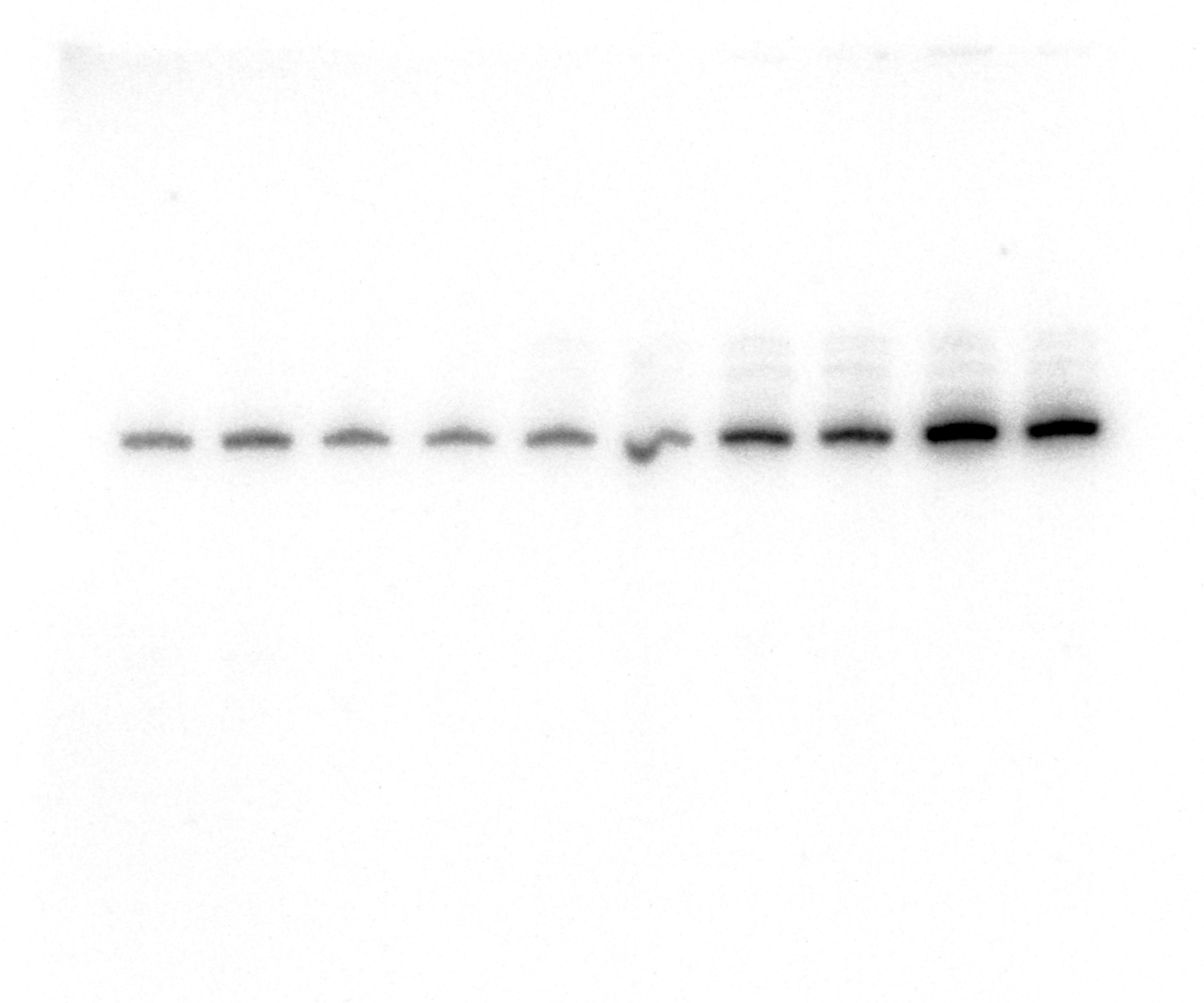

Supplement: Supplementary file 8 — Source data Fig. 2 [file 44318_2024_265_MOESM8_ESM.zip › SD_Figure2/Source_data_Figure2E/northern_Thr_UGU_8.tiff]

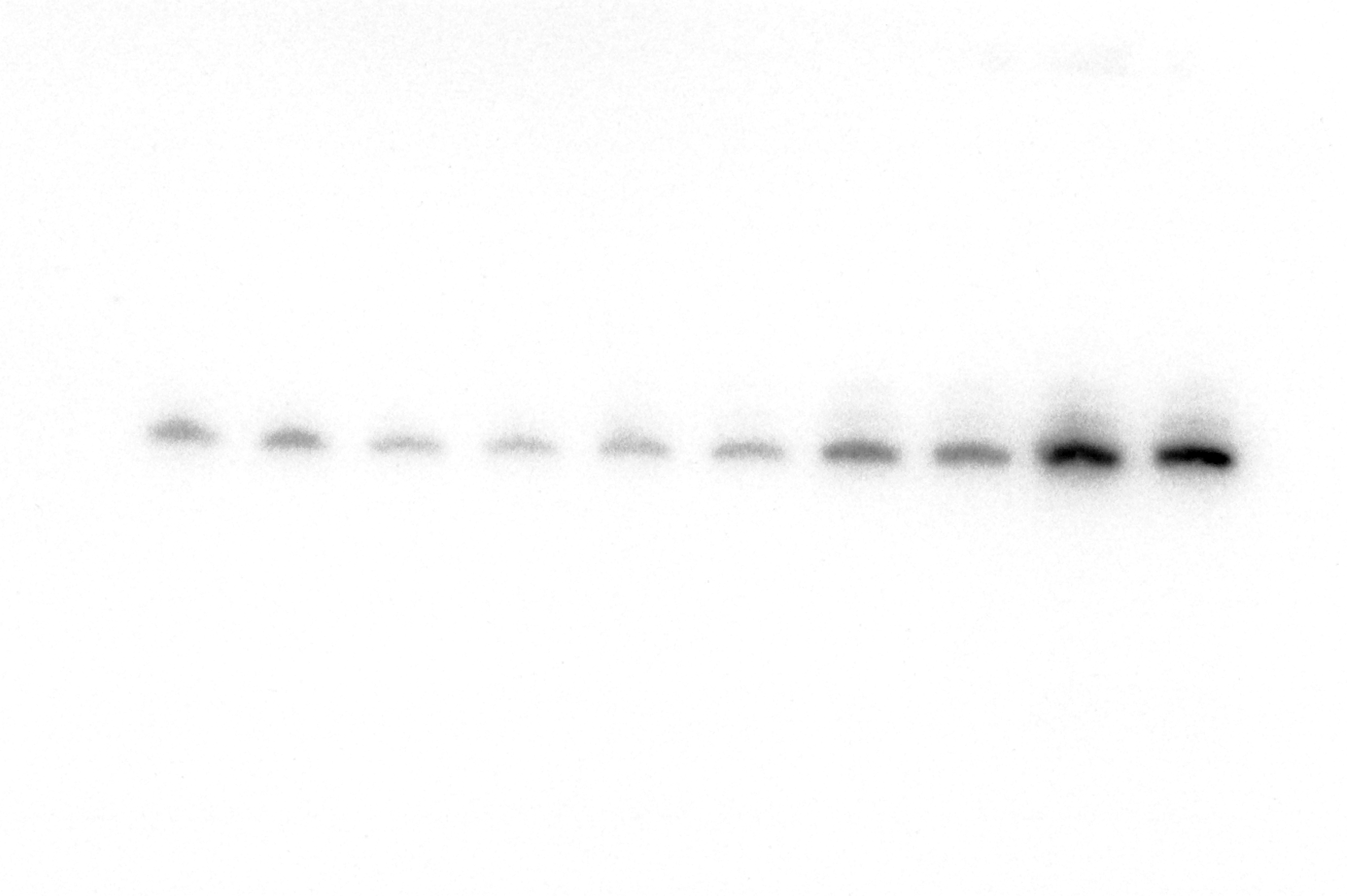

Supplement: Supplementary file 8 — Source data Fig. 2 [file 44318_2024_265_MOESM8_ESM.zip › SD_Figure2/Source_data_Figure2E/northern_Ser_UGA_19.tiff]

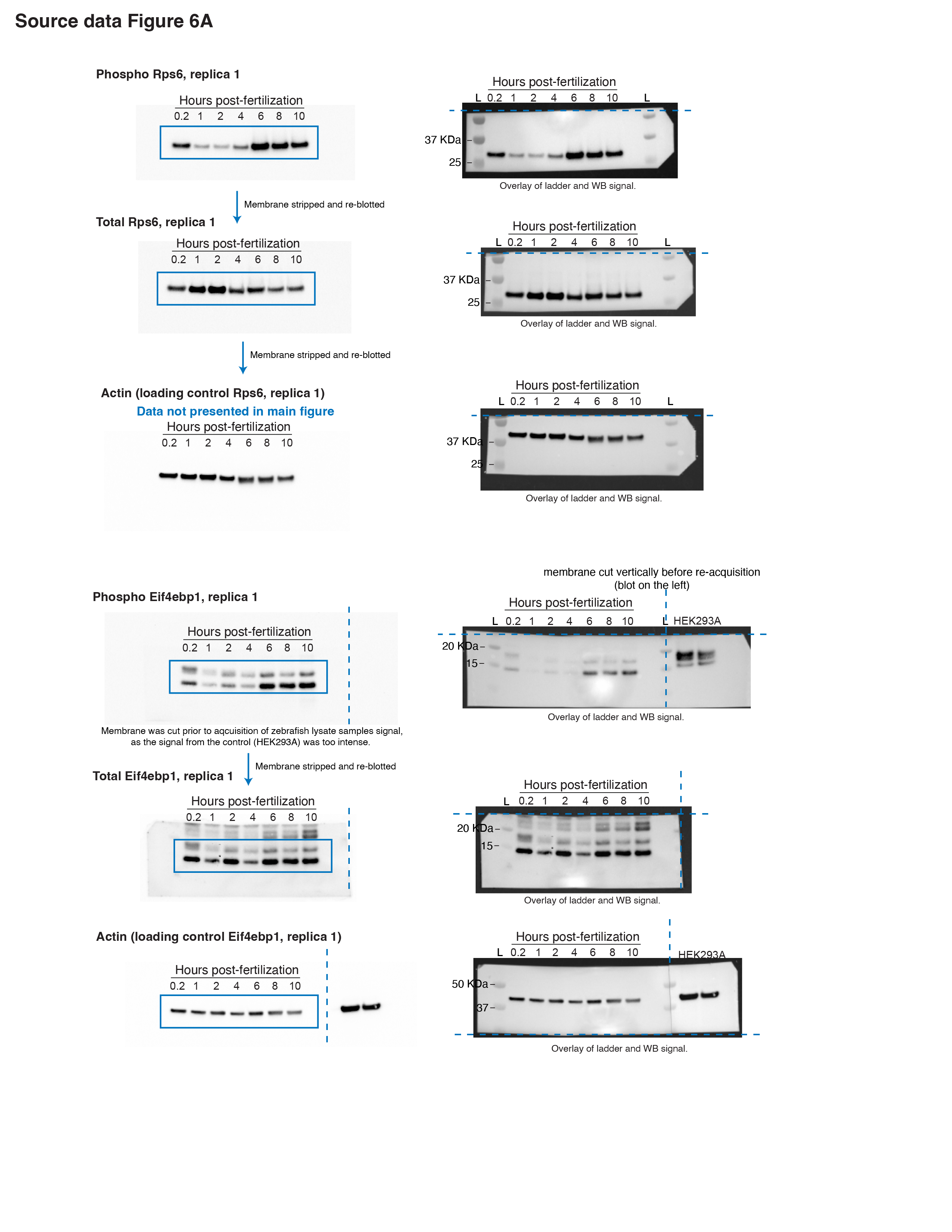

Supplement: Supplementary file 10 — Source data Fig. 6 [file 44318_2024_265_MOESM10_ESM.zip › SD_Figure6/Source_data_Figure6A/Source_data_Figure6A_WBs.png]

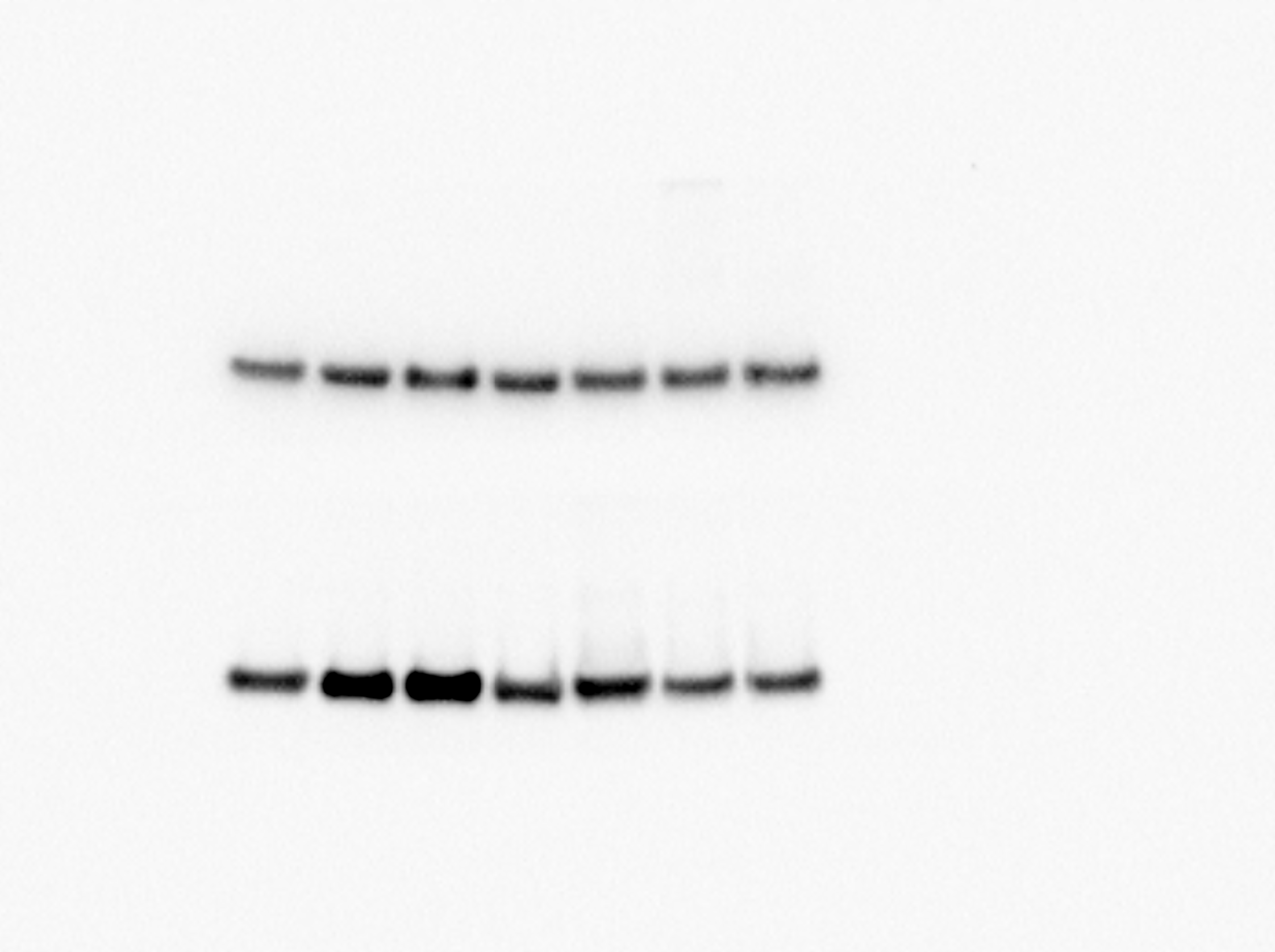

Supplement: Supplementary file 10 — Source data Fig. 6 [file 44318_2024_265_MOESM10_ESM.zip › SD_Figure6/Source_data_Figure6A/S6RP_total_rep1.tif]

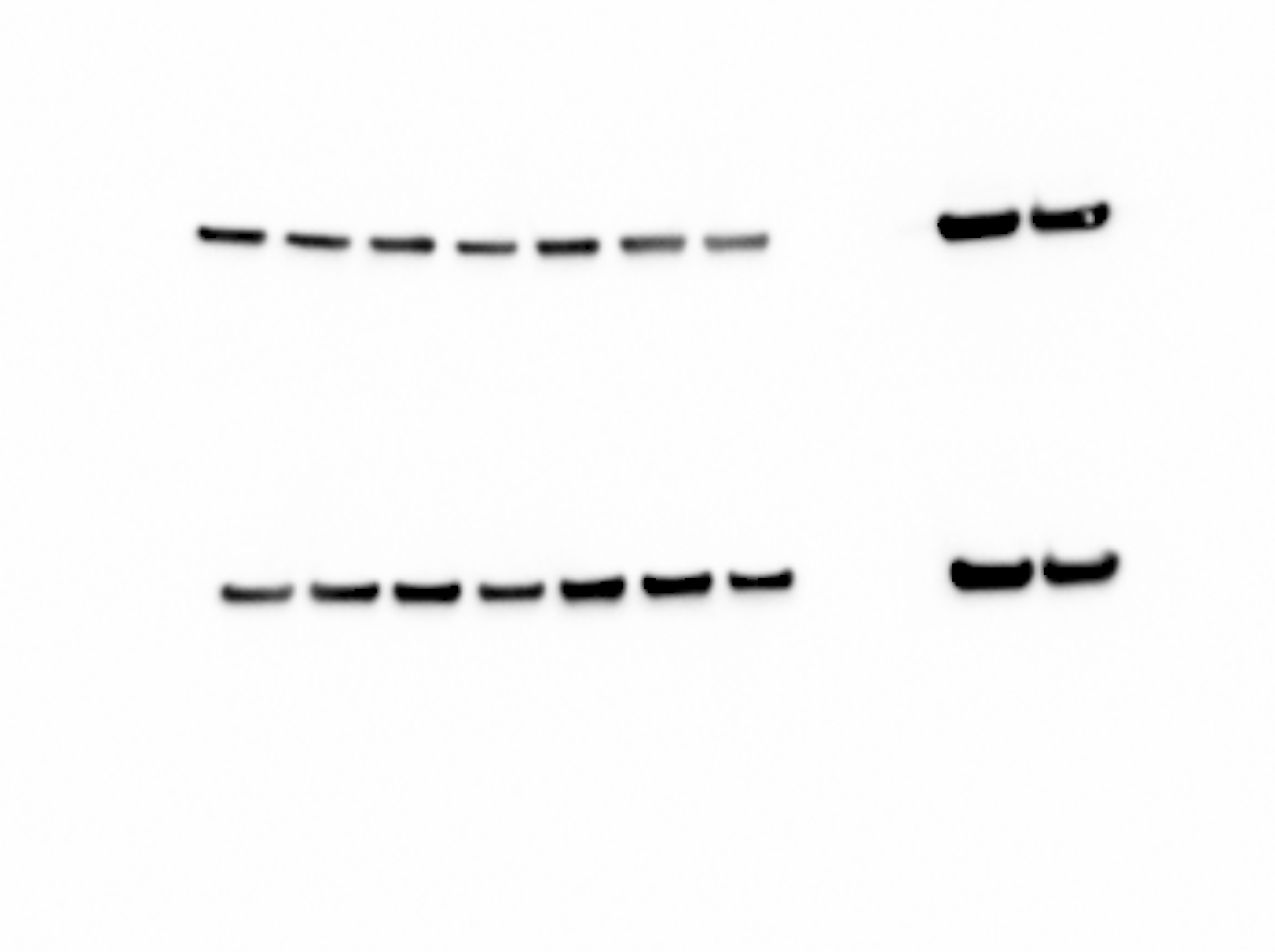

Supplement: Supplementary file 10 — Source data Fig. 6 [file 44318_2024_265_MOESM10_ESM.zip › SD_Figure6/Source_data_Figure6A/Actin_rep1.tif]

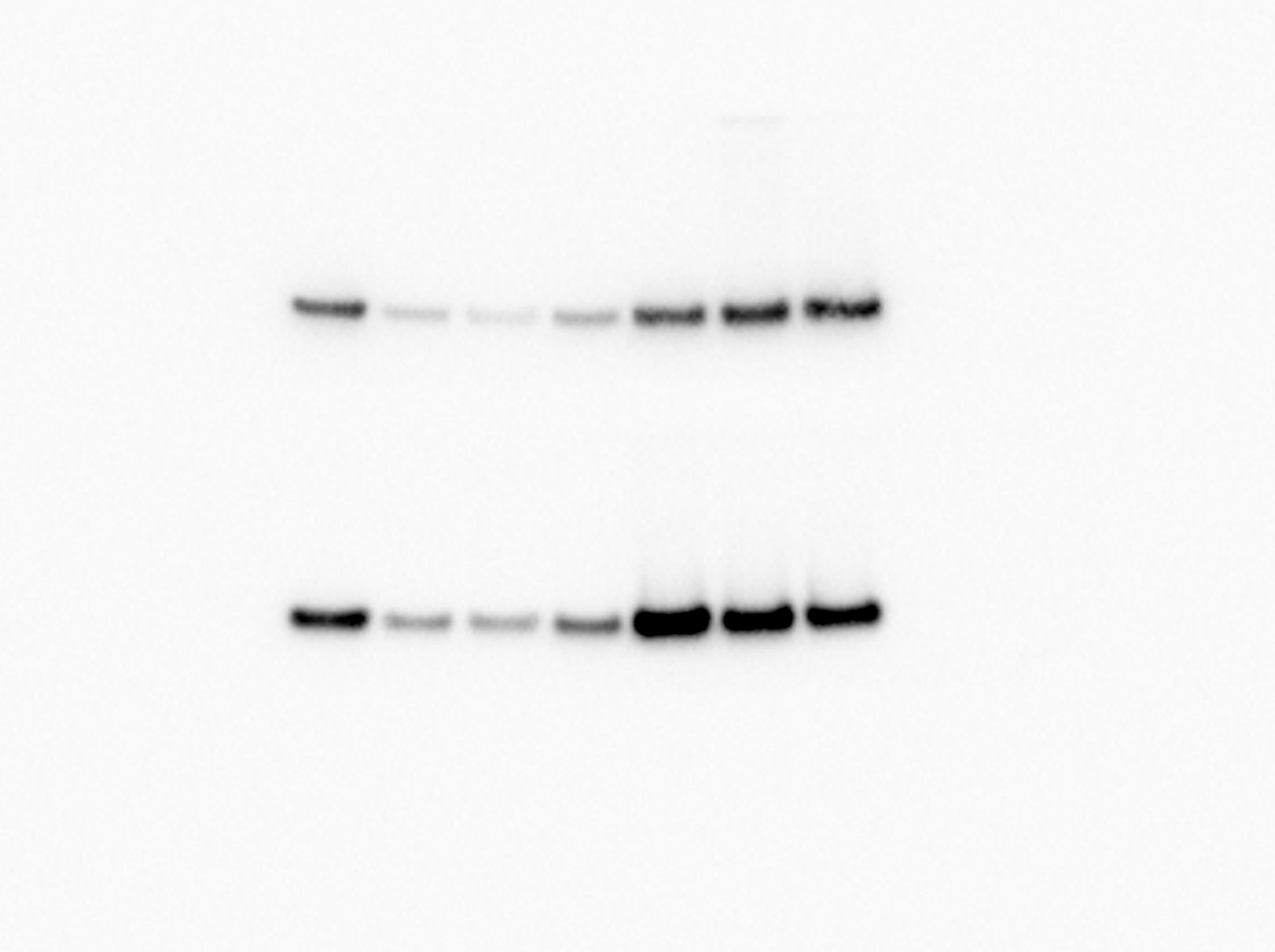

Supplement: Supplementary file 10 — Source data Fig. 6 [file 44318_2024_265_MOESM10_ESM.zip › SD_Figure6/Source_data_Figure6A/S6RP_phospho_rep1.tif]

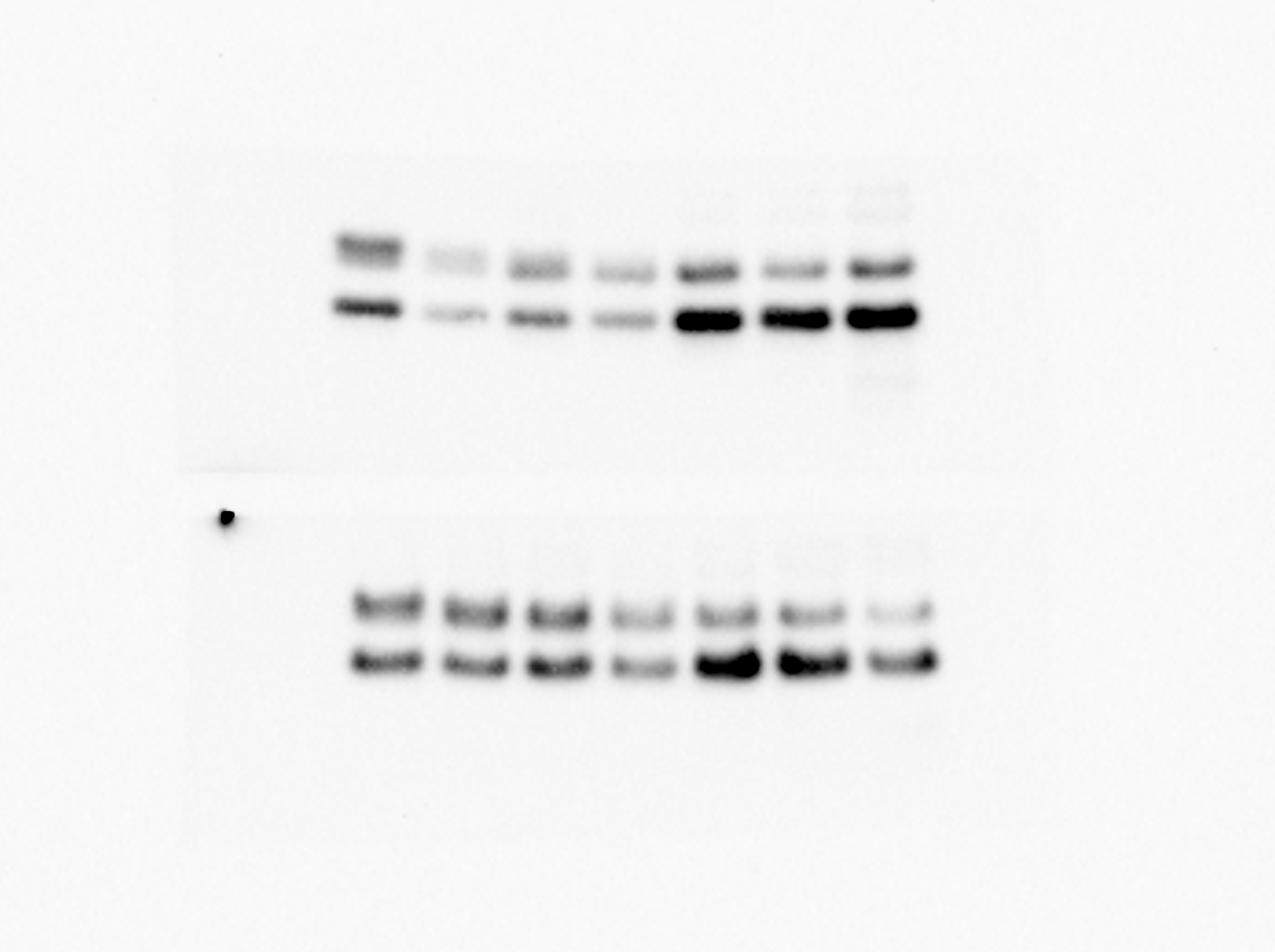

Supplement: Supplementary file 10 — Source data Fig. 6 [file 44318_2024_265_MOESM10_ESM.zip › SD_Figure6/Source_data_Figure6A/4EBP1_phospho_rep1.tif]

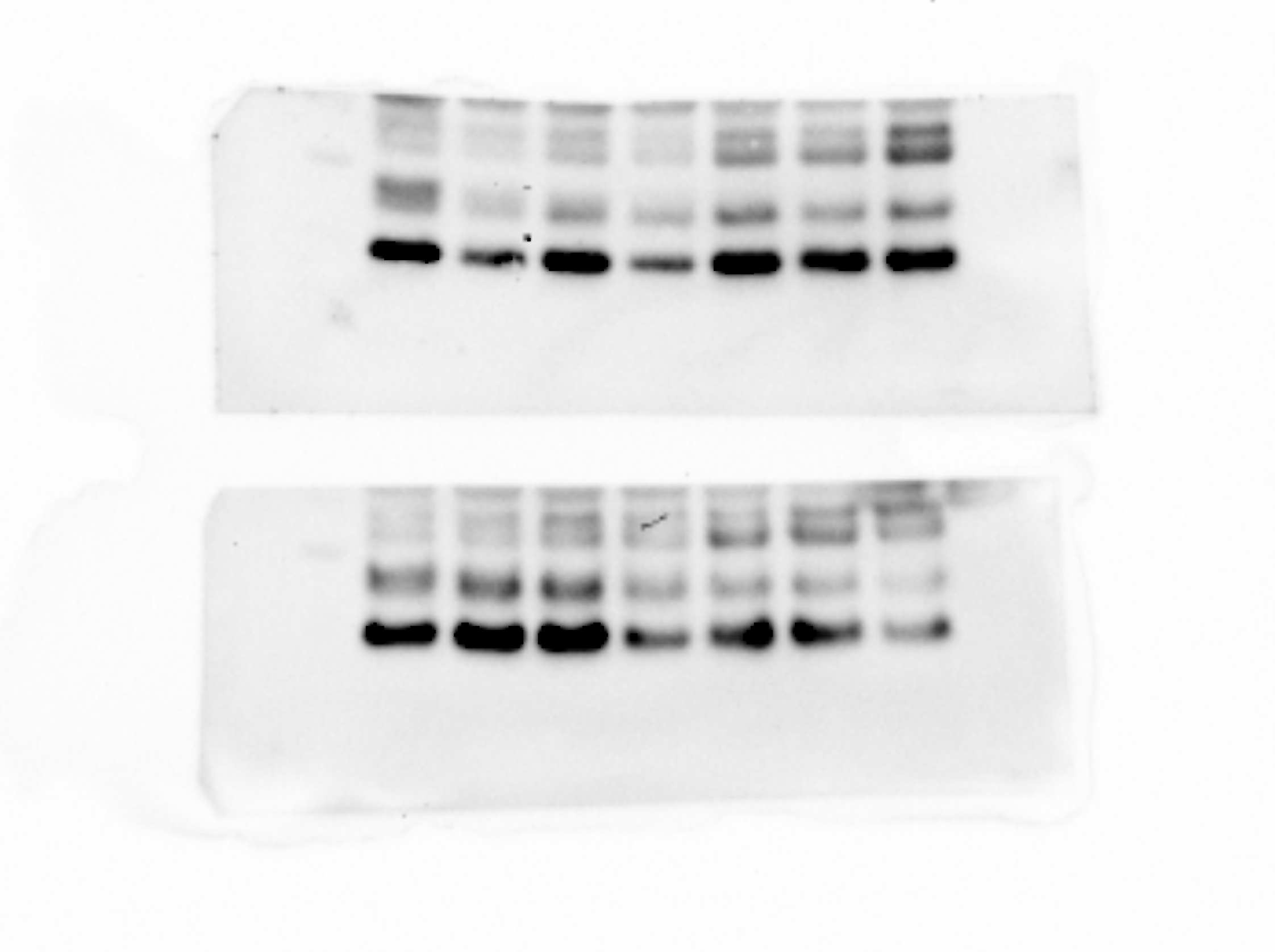

Supplement: Supplementary file 10 — Source data Fig. 6 [file 44318_2024_265_MOESM10_ESM.zip › SD_Figure6/Source_data_Figure6A/4EBP1_total_rep1.tif]

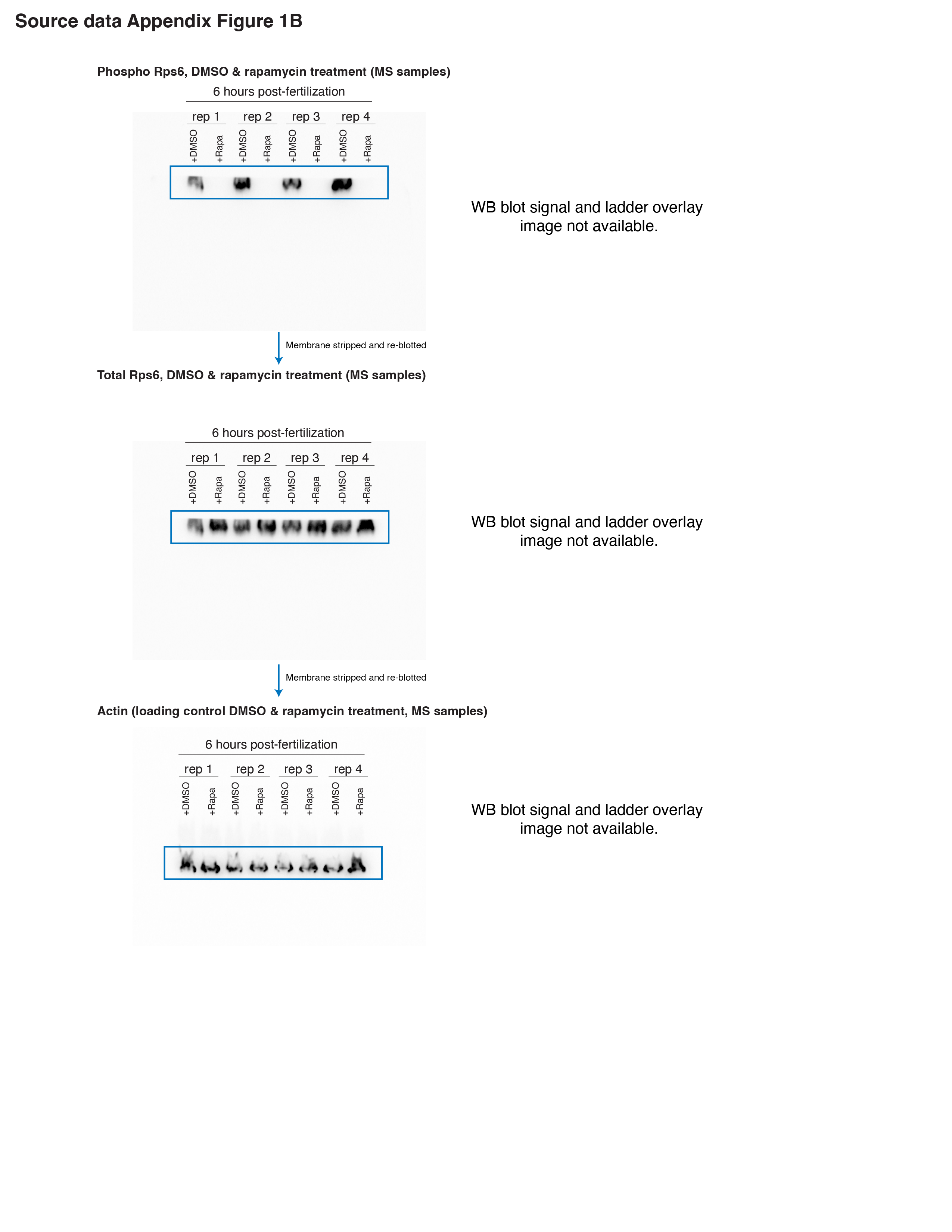

Supplement: Supplementary file 11 — Appendix Source Data [file 44318_2024_265_MOESM11_ESM.zip › SD_AppendixFigure1/Source_data_AppendixFigure1B/Source_data_AppendixFigure1B_WBs.png]

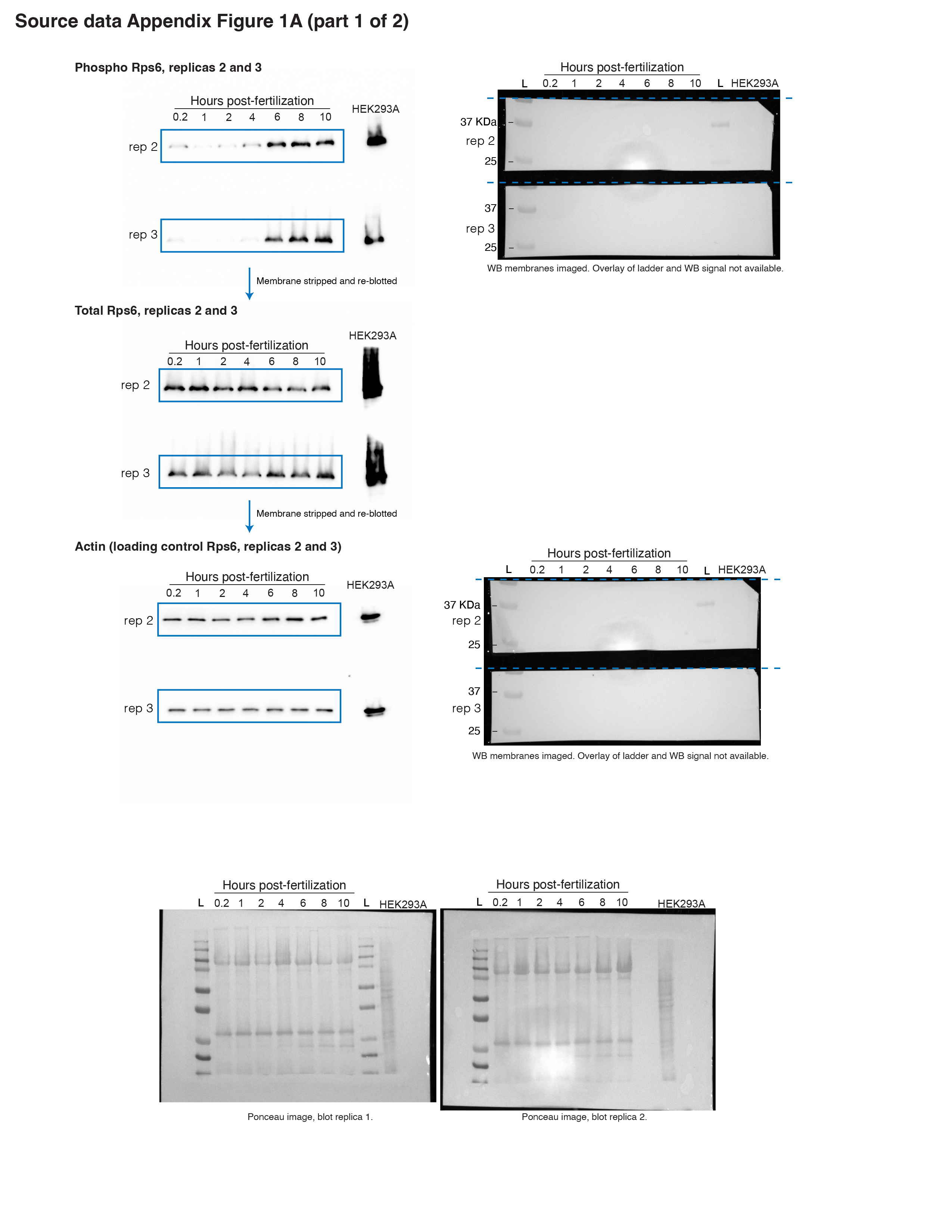

Supplement: Supplementary file 11 — Appendix Source Data [file 44318_2024_265_MOESM11_ESM.zip › SD_AppendixFigure1/Source_data_AppendixFigure1A/Source_data_AppendixFigure1A_WBs-01.png]

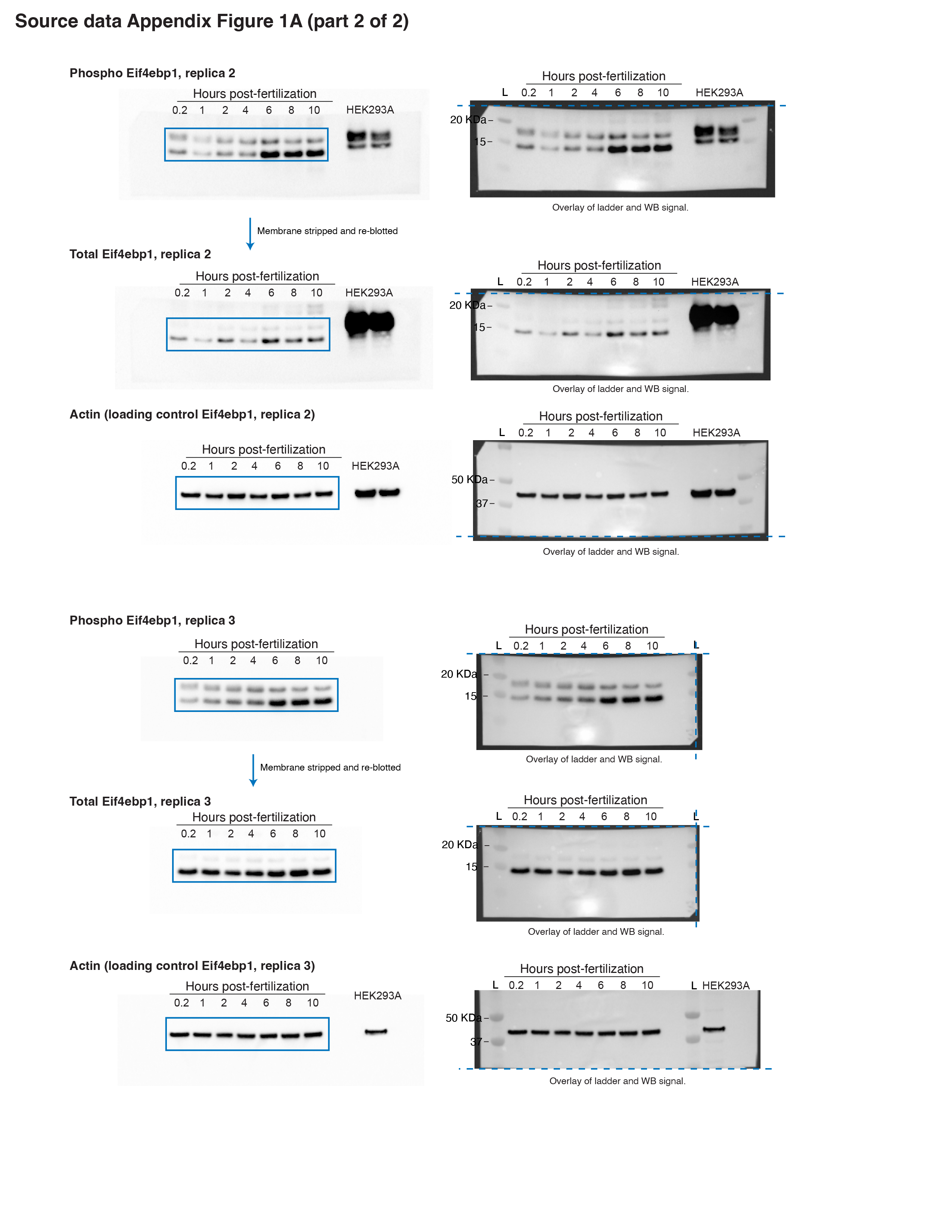

Supplement: Supplementary file 11 — Appendix Source Data [file 44318_2024_265_MOESM11_ESM.zip › SD_AppendixFigure1/Source_data_AppendixFigure1A/Source_data_AppendixFigure1A_WBs-02.png]

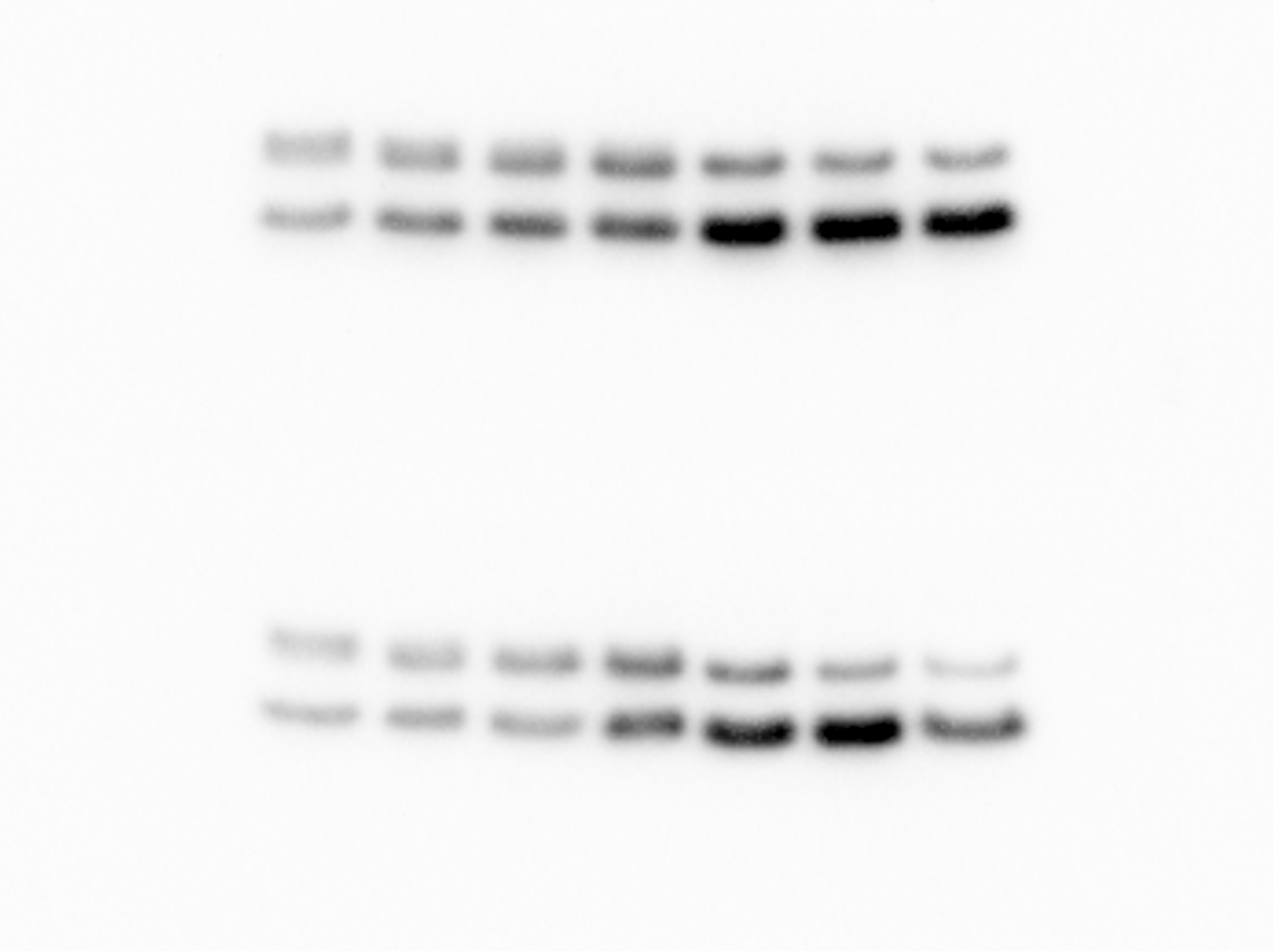

Supplement: Supplementary file 11 — Appendix Source Data [file 44318_2024_265_MOESM11_ESM.zip › SD_AppendixFigure1/Source_data_AppendixFigure1A/4EBP1_phospho_rep3.tif]

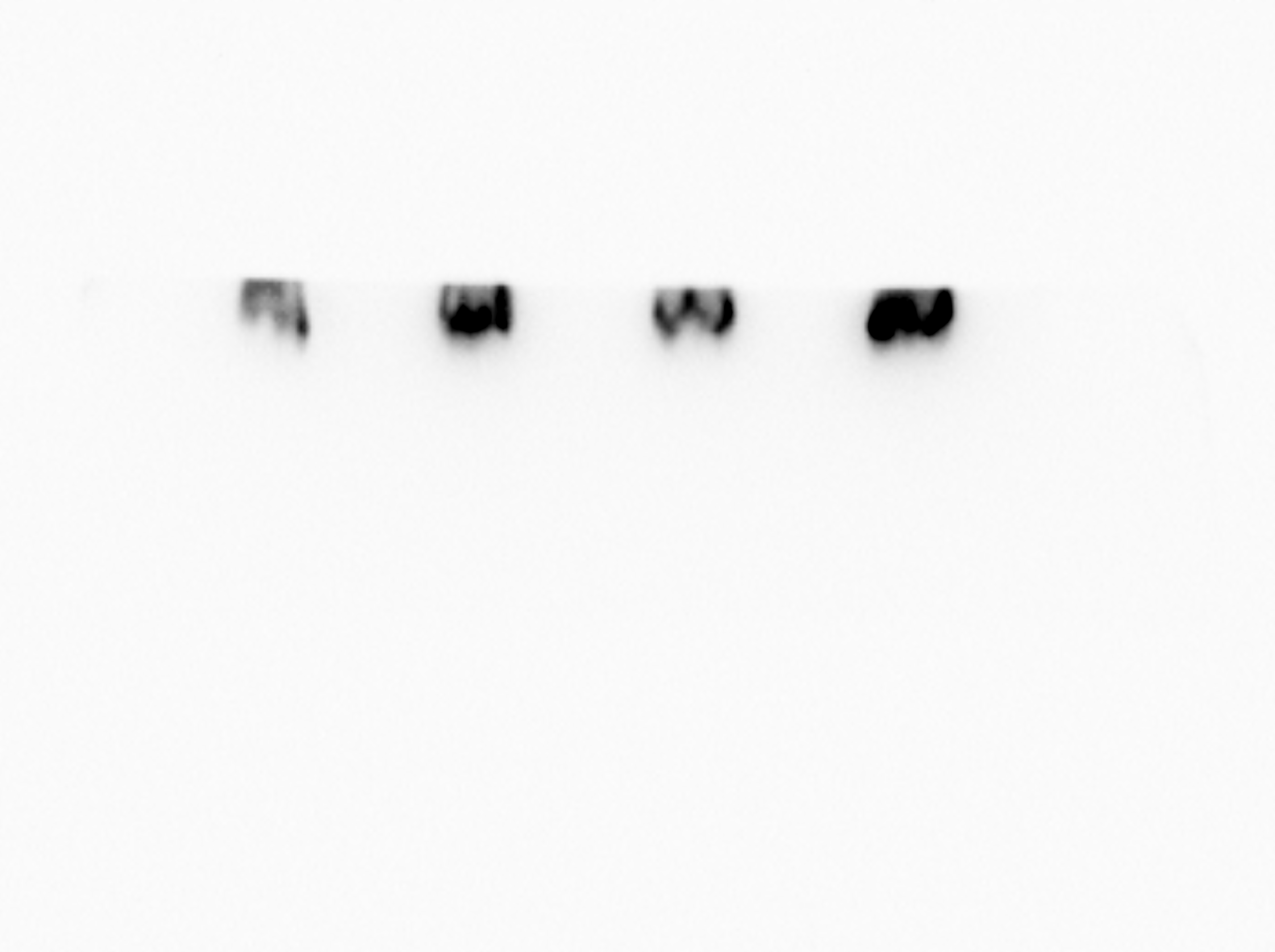

Supplement: Supplementary file 11 — Appendix Source Data [file 44318_2024_265_MOESM11_ESM.zip › SD_AppendixFigure1/Source_data_AppendixFigure1B/RPS6_phospho_rapa.tif]

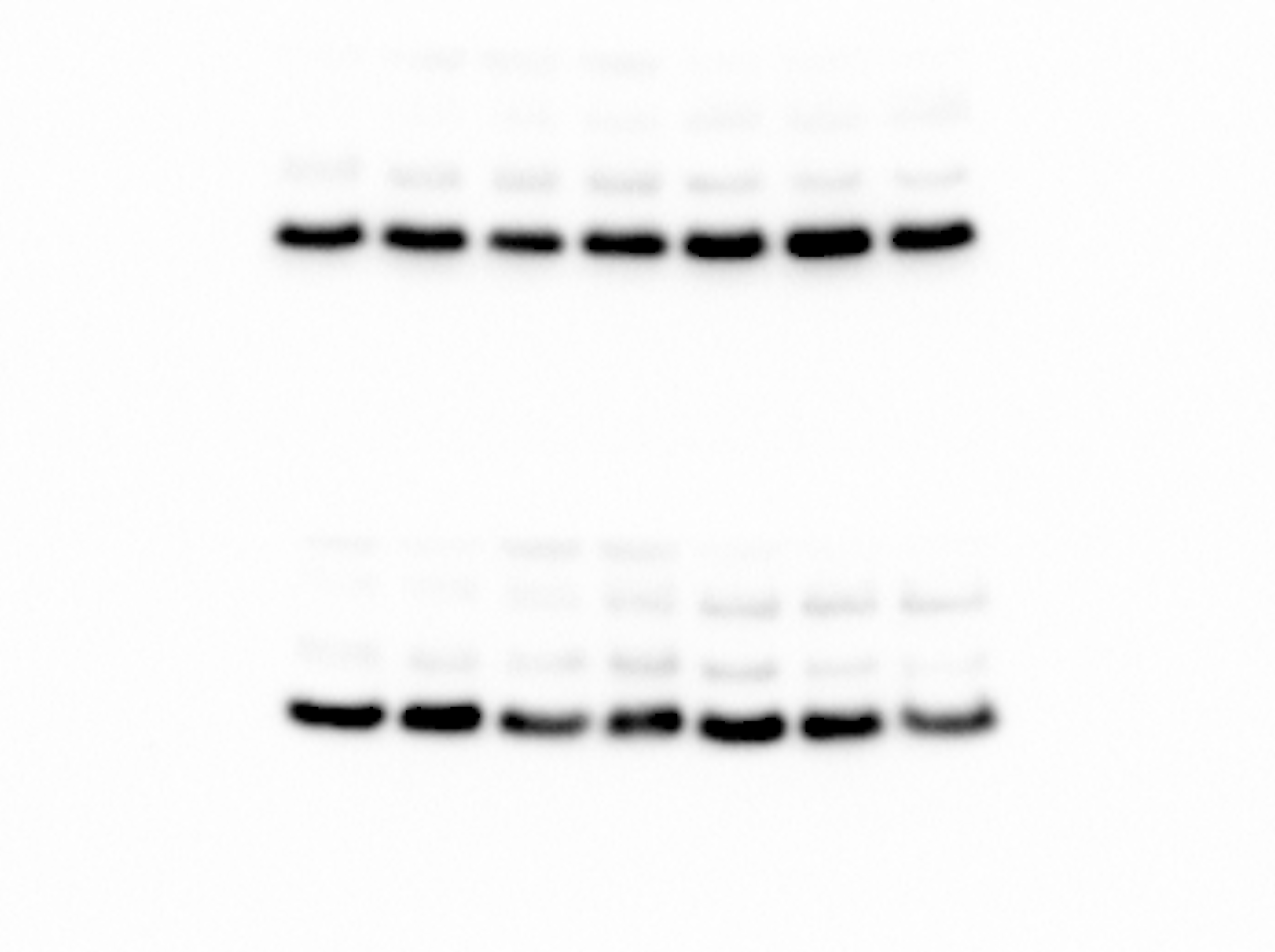

Supplement: Supplementary file 11 — Appendix Source Data [file 44318_2024_265_MOESM11_ESM.zip › SD_AppendixFigure1/Source_data_AppendixFigure1A/4EBP1_total_rep3.tif]

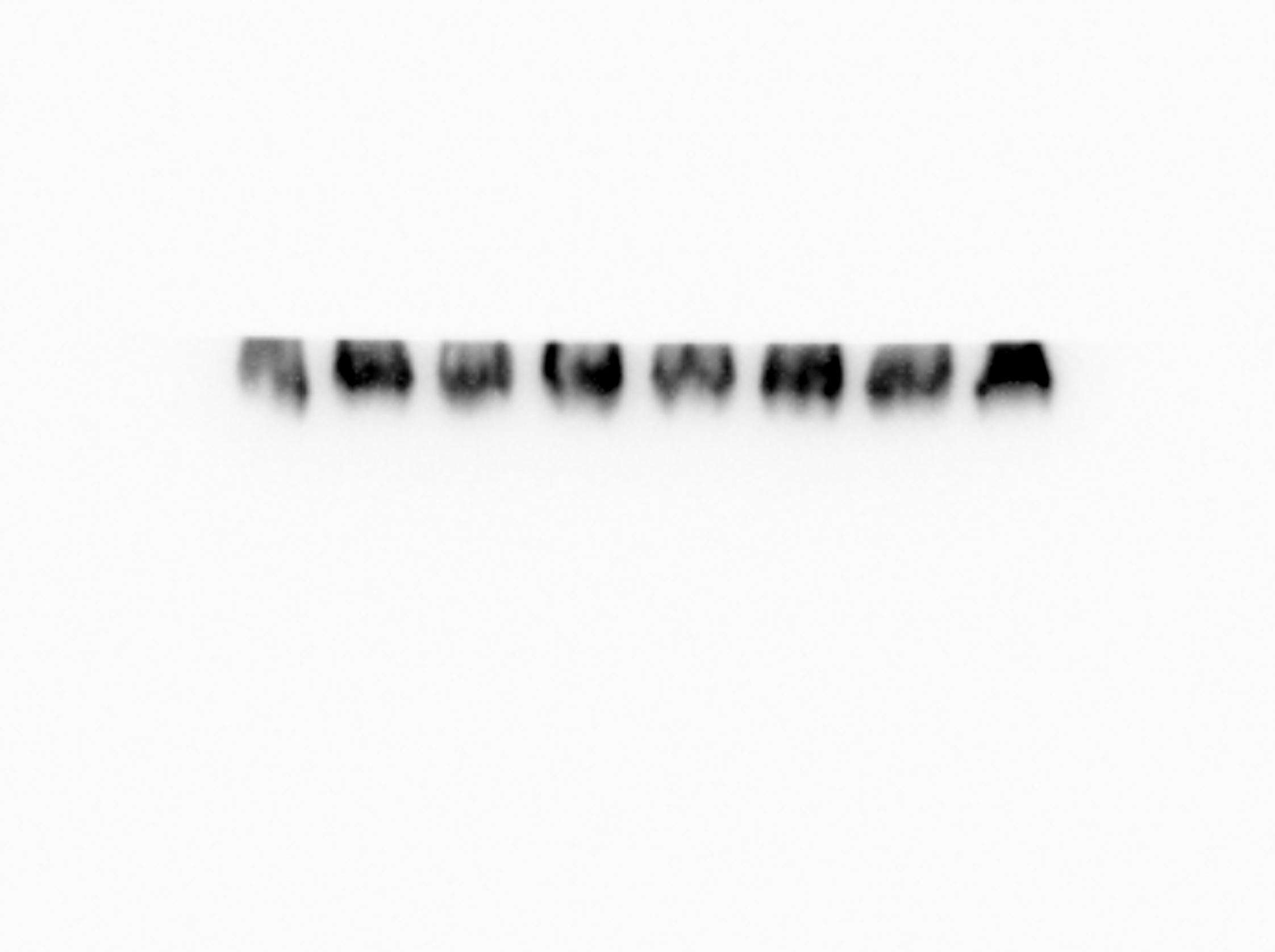

Supplement: Supplementary file 11 — Appendix Source Data [file 44318_2024_265_MOESM11_ESM.zip › SD_AppendixFigure1/Source_data_AppendixFigure1B/RPS6_total_rapa.tif]

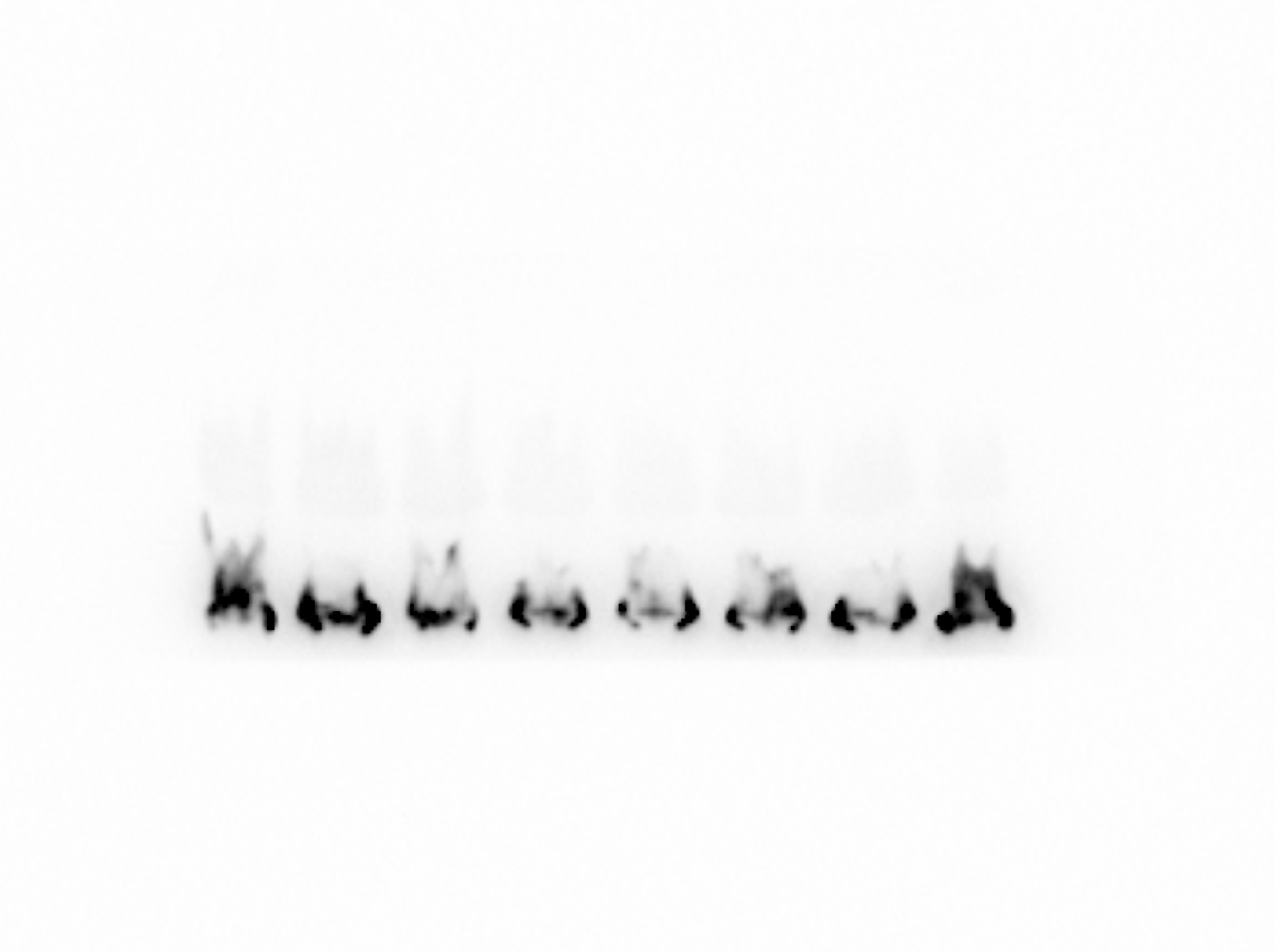

Supplement: Supplementary file 11 — Appendix Source Data [file 44318_2024_265_MOESM11_ESM.zip › SD_AppendixFigure1/Source_data_AppendixFigure1B/Actin_rapa.tif]

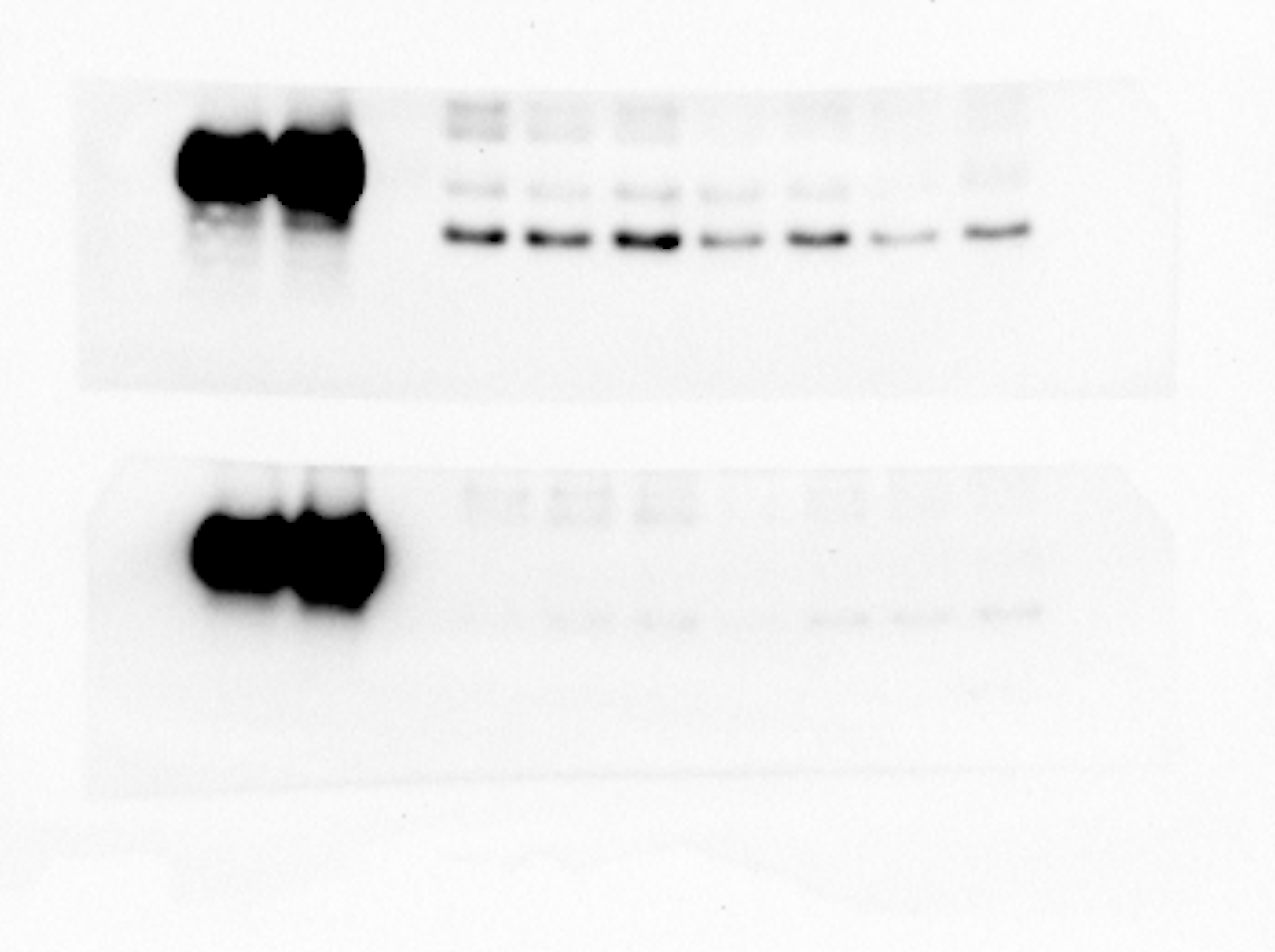

Supplement: Supplementary file 11 — Appendix Source Data [file 44318_2024_265_MOESM11_ESM.zip › SD_AppendixFigure1/Source_data_AppendixFigure1A/4EBP1_total_rep2.tif]

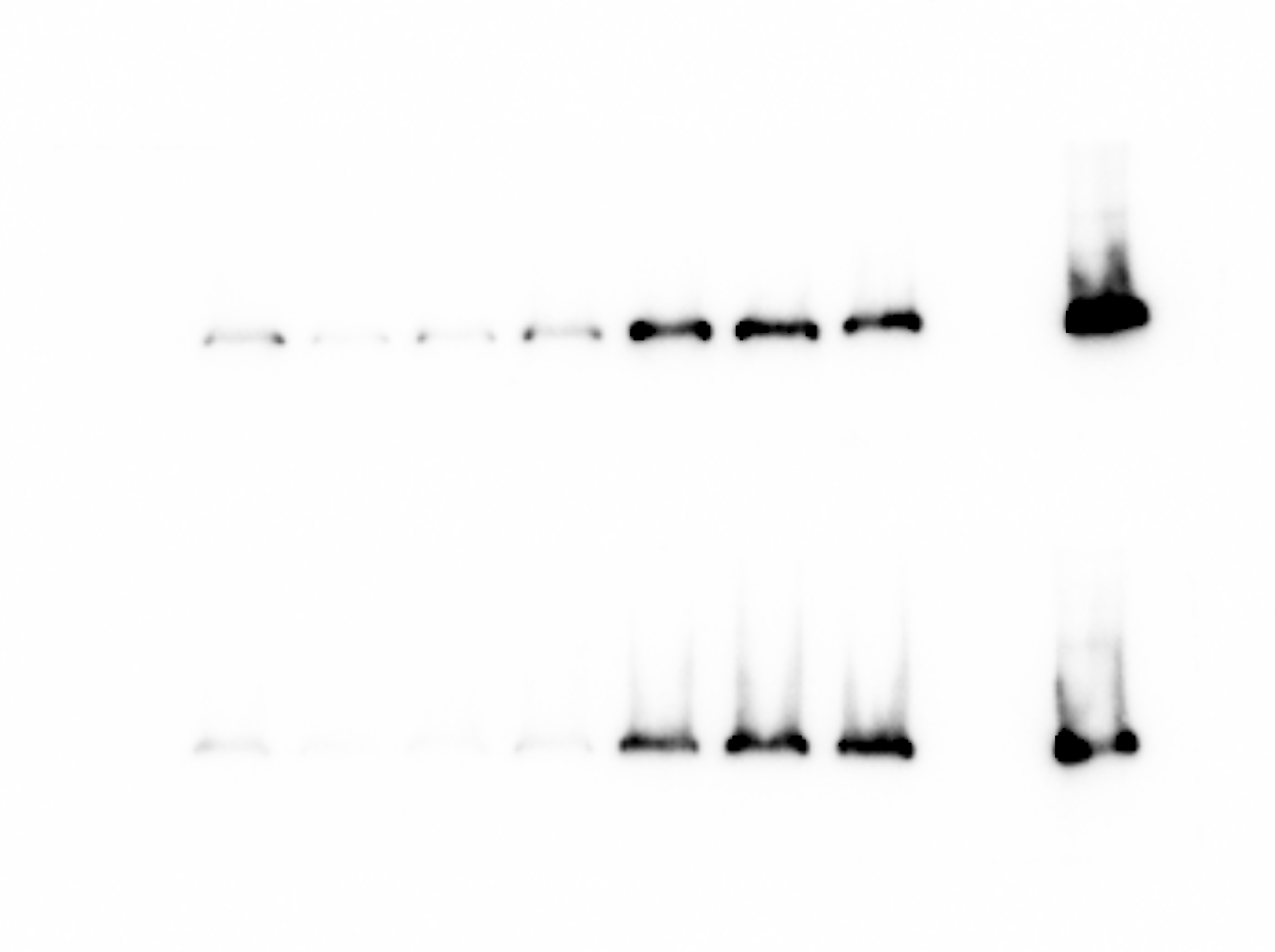

Supplement: Supplementary file 11 — Appendix Source Data [file 44318_2024_265_MOESM11_ESM.zip › SD_AppendixFigure1/Source_data_AppendixFigure1A/SRP6_phospho_reps2and3.tif]

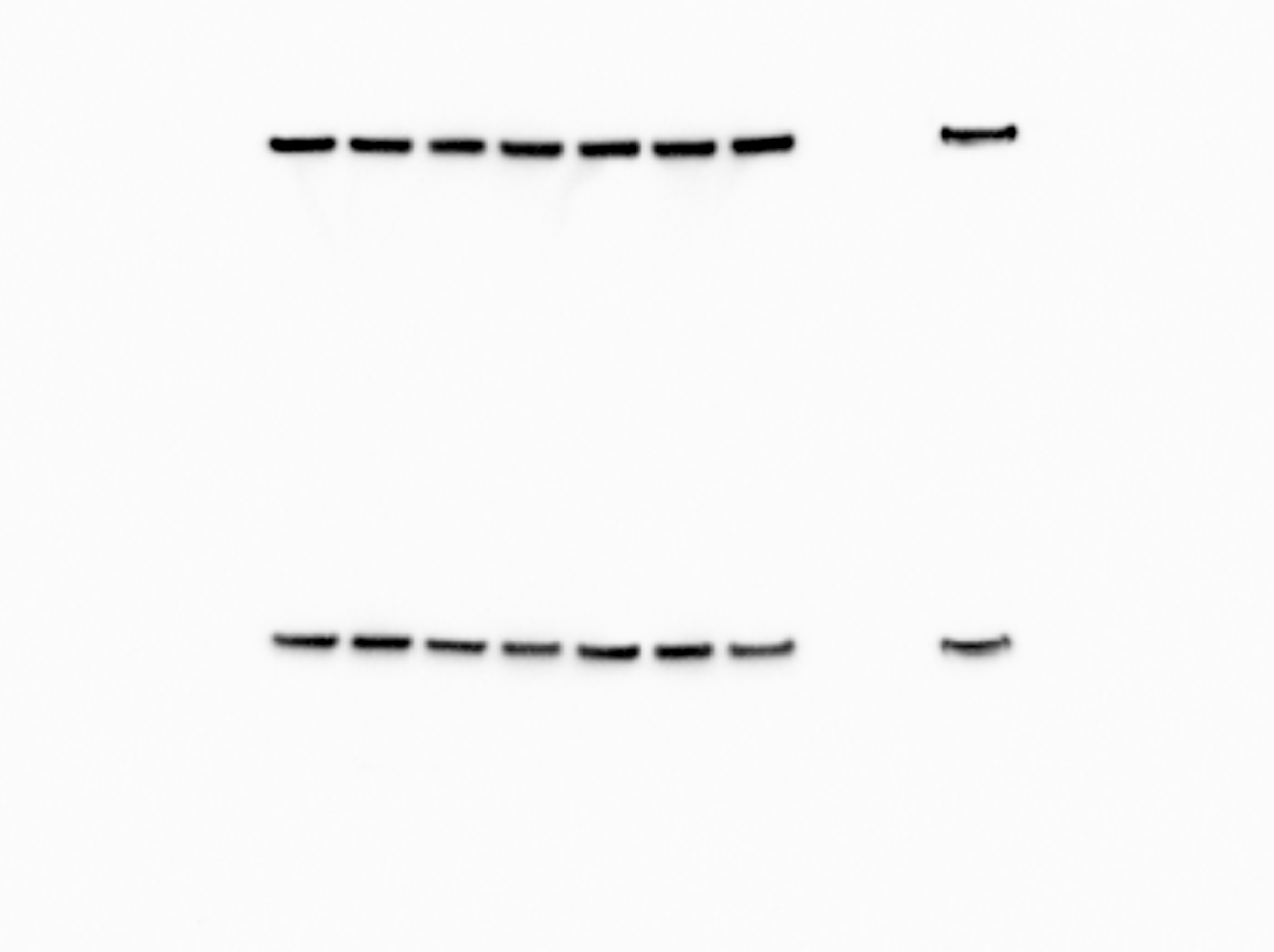

Supplement: Supplementary file 11 — Appendix Source Data [file 44318_2024_265_MOESM11_ESM.zip › SD_AppendixFigure1/Source_data_AppendixFigure1A/Actin_eif4ebp1_loading_control_rep3.tif]

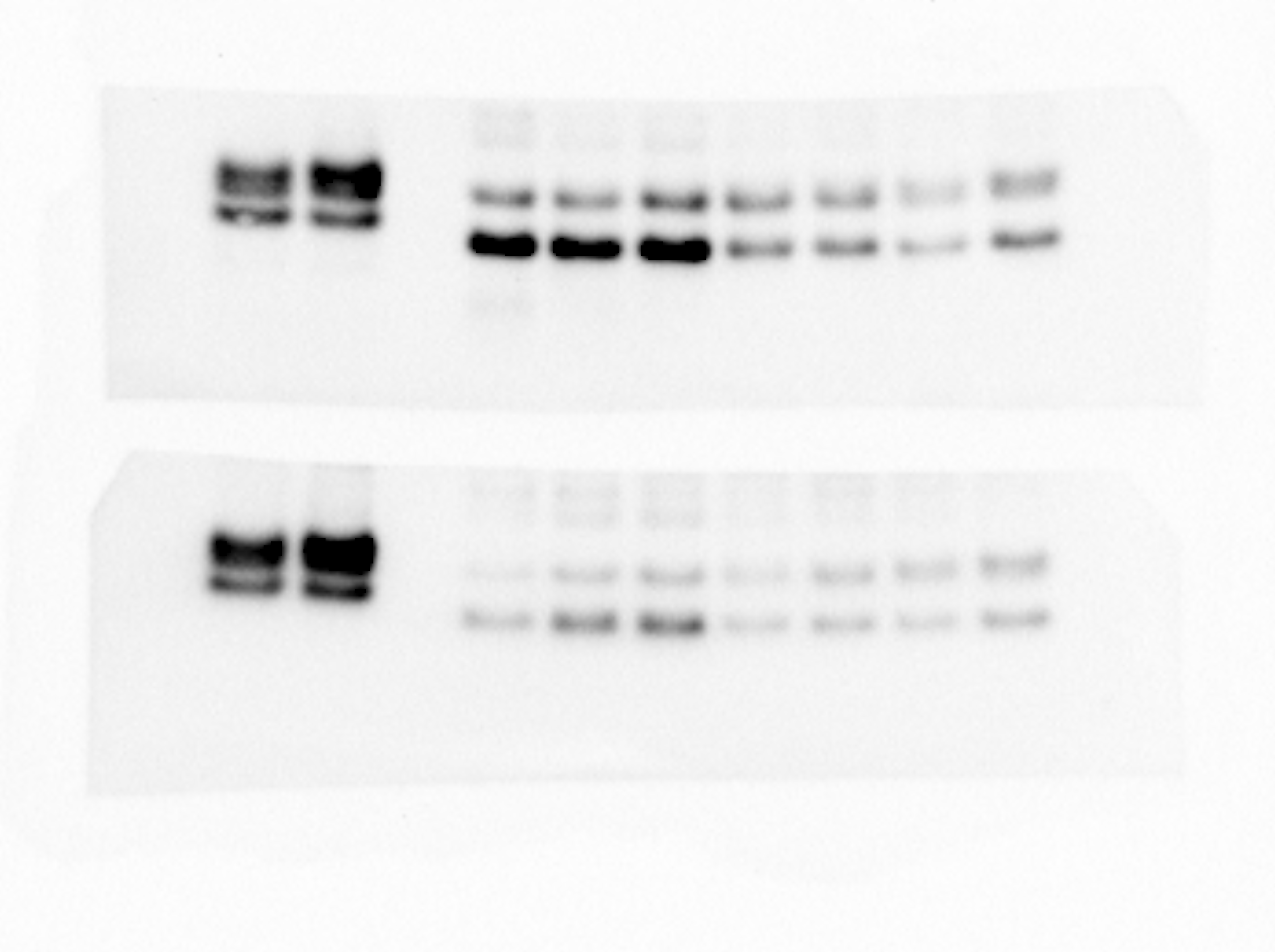

Supplement: Supplementary file 11 — Appendix Source Data [file 44318_2024_265_MOESM11_ESM.zip › SD_AppendixFigure1/Source_data_AppendixFigure1A/4EBP1_phospho_rep2.tif]

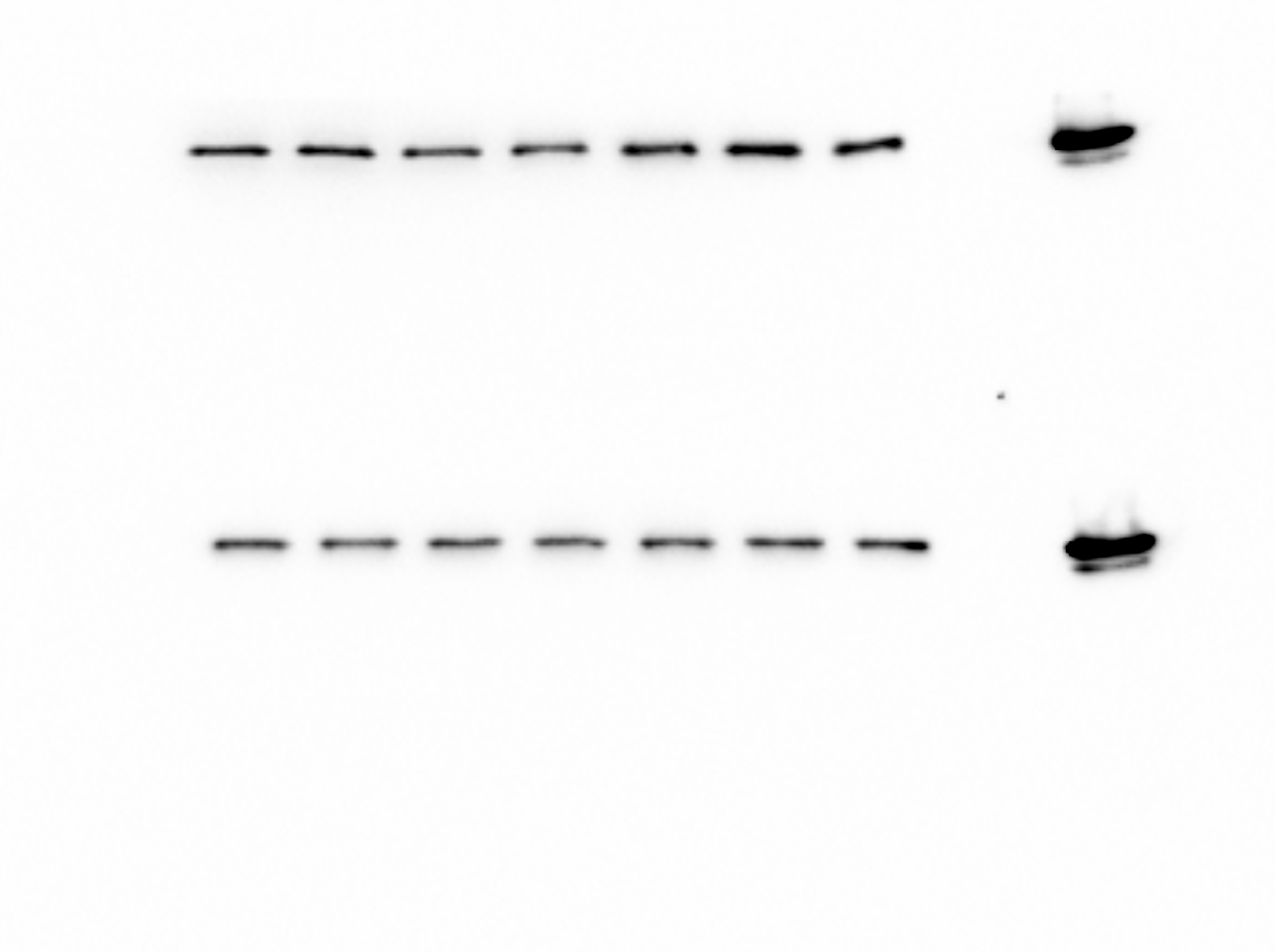

Supplement: Supplementary file 11 — Appendix Source Data [file 44318_2024_265_MOESM11_ESM.zip › SD_AppendixFigure1/Source_data_AppendixFigure1A/Actin_rps6_loading_control_reps2and3.tif]

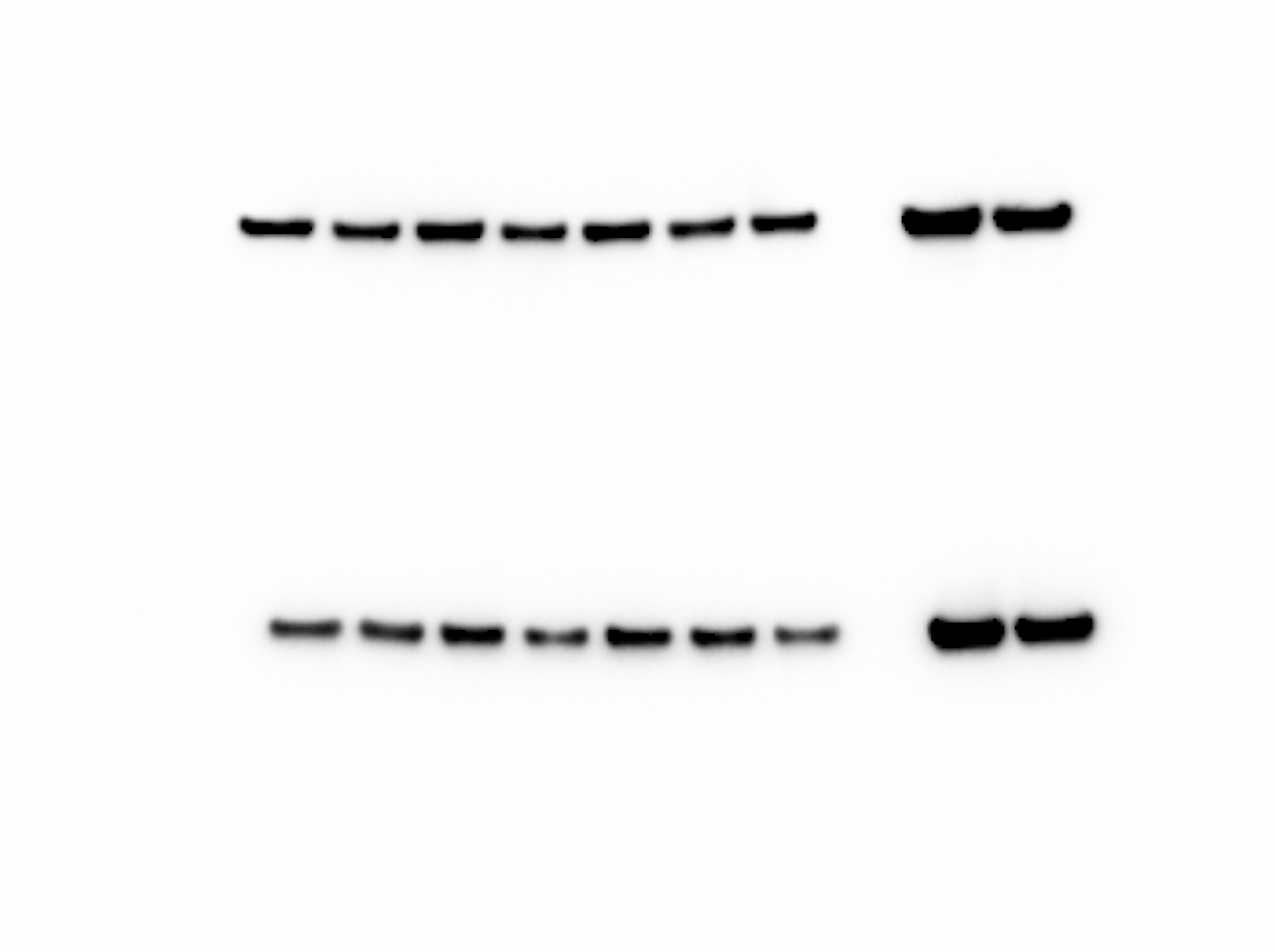

Supplement: Supplementary file 11 — Appendix Source Data [file 44318_2024_265_MOESM11_ESM.zip › SD_AppendixFigure1/Source_data_AppendixFigure1A/Actin_eif4ebp1_loading_control_rep2.tif]

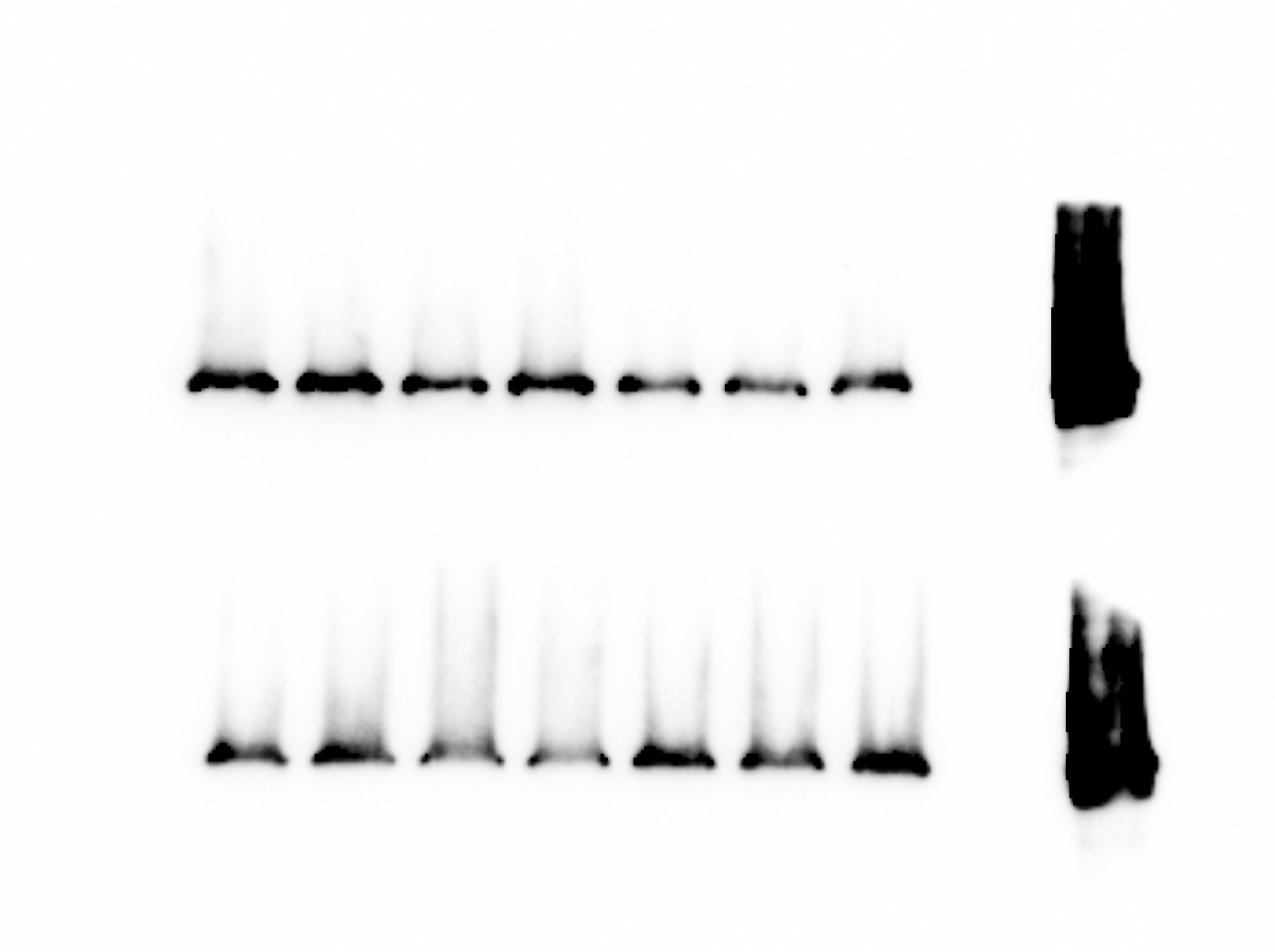

Supplement: Supplementary file 11 — Appendix Source Data [file 44318_2024_265_MOESM11_ESM.zip › SD_AppendixFigure1/Source_data_AppendixFigure1A/SRP6_total_reps2and3.tif]
