# Supplementary material for: The dynamics and functional impact of tRNA repertoires during early embryogenesis in zebrafish
Source: EMBO J. 2024 Oct 14;43(22):19. doi: 10.1038/s44318-024-00265-4 (PMC11574265; doi:10.1038/s44318-024-00265-4)
Supplement: Supplementary file 9 — Source data Fig. 3 [file 44318_2024_265_MOESM9_ESM.zip › Source_data_Figure3A/README_source_data_Figure3A.rtf]

The file “polysome_prfiles_Fig3A.csv” corresponds to the file outputted by the polysome profiling machine when the data presented in figure 3A was acquired. The raw data is plotted in the figure, with an imposed y-axis limit (depicting absorbance values ranging from 0 to 0.6) for clearer data visualization.
