# Supplementary material for: The dynamics and functional impact of tRNA repertoires during early embryogenesis in zebrafish
Source: EMBO J. 2024 Oct 14;43(22):19. doi: 10.1038/s44318-024-00265-4 (PMC11574265; doi:10.1038/s44318-024-00265-4)
Supplement: Supplementary file 10 — Source data Fig. 6 [file 44318_2024_265_MOESM10_ESM.zip › SD_Figure6/Source_data_Figure6D/README_source_data_Figure6D.rtf]

The excel file contains mass spectrometry data (LFQ values, a.u.) for Maf1a and Maf1b proteins of wild-type zebrafish embryos at different stages of development: 3, 6 and 10 hours post-fertilization (hpf). For each time-point, 5 technical replicates were acquired and quantified. 
