# Supplementary material for: The dynamics and functional impact of tRNA repertoires during early embryogenesis in zebrafish
Source: EMBO J. 2024 Oct 14;43(22):19. doi: 10.1038/s44318-024-00265-4 (PMC11574265; doi:10.1038/s44318-024-00265-4)
Supplement: Supplementary file 10 — Source data Fig. 6 [file 44318_2024_265_MOESM10_ESM.zip › SD_Figure6/Source_data_Figure6A/README_source_data_Figure6A.rtfd/TXT.rtf]

Below is the description of the WB raw images presented in figure 6A.Western blot phospho rps6 replica 1: S6RP_phospho_rep1.tifWestern blot total rps6 replica 1: S6RP_total_rep1.tifWestern blot phospho eif4ebp1 replica 1: 4EBP1_phospho_rep1.tifWestern blot total eif4ebp1 replica 1: 4EBP1_total_rep1.tifWestern blot loading control replica 1: Actin_rep1.tif
