# Supplementary material for: The dynamics and functional impact of tRNA repertoires during early embryogenesis in zebrafish
Source: EMBO J. 2024 Oct 14;43(22):19. doi: 10.1038/s44318-024-00265-4 (PMC11574265; doi:10.1038/s44318-024-00265-4)
Supplement: Supplementary file 11 — Appendix Source Data [file 44318_2024_265_MOESM11_ESM.zip › SD_AppendixFigure1/Source_data_AppendixFigure1A/README_source_data_AppendixFigure1A.rtf]

Below is the description of the WB raw images presented in Appendix figure 1A.Western blot phospho rps6 replicas 2 and 3: SRP6_phospho_reps2and3.tifWestern blot total rps6 replicas 2 and 3: SRP6_total_reps2and3.tifWestern blot loading control rps6 replicas 2 and 3: Actin_rps6_loading_control_reps2and3.tifWestern blot phospho eif4ebp1 replica 2: 4EBP1_phospho_rep2.tifWestern blot total eif4ebp1 replica 2: 4EBP1_total_rep2.tifWestern blot loading control eif4ebp1 replica 2: Actin_eif4ebp1_loading_control_rep2.tifWestern blot phospho eif4ebp1 replica 3: 4EBP1_phospho_rep3.tifWestern blot total eif4ebp1 replica 3: 4EBP1_total_rep3.tifWestern blot loading control eif4ebp1 replica 3: Actin_eif4ebp1_loading_control_rep3.tif
