# Supplementary material for: The dynamics and functional impact of tRNA repertoires during early embryogenesis in zebrafish
Source: EMBO J. 2024 Oct 14;43(22):19. doi: 10.1038/s44318-024-00265-4 (PMC11574265; doi:10.1038/s44318-024-00265-4)
Supplement: Supplementary file 11 — Appendix Source Data [file 44318_2024_265_MOESM11_ESM.zip › SD_AppendixFigure1/Source_data_AppendixFigure1B/README_source_data_AppendixFigure1B.rtf]

Below is the description of the WB raw images presented in Appendix figure 1B.Western blot phospho rps6 rapamycin and control DMSO treated embryos, reps 1-4: S6RP_phospho_rapa.tif Western blot total rps6 rps6 rapamycin and control DMSO treated embryos, reps 1-4: S6RP_total_rapa.tif Western blot loading control rps6 rapamycin and control DMSO treated embryos, reps 1-4: Actin_rapa.tif 
