# Supplementary material for: The dynamics and functional impact of tRNA repertoires during early embryogenesis in zebrafish
Source: EMBO J. 2024 Oct 14;43(22):19. doi: 10.1038/s44318-024-00265-4 (PMC11574265; doi:10.1038/s44318-024-00265-4)
Supplement: Supplementary file 12 — Expanded View Figures [file 44318_2024_265_MOESM12_ESM.pdf]

Expanded View Figures

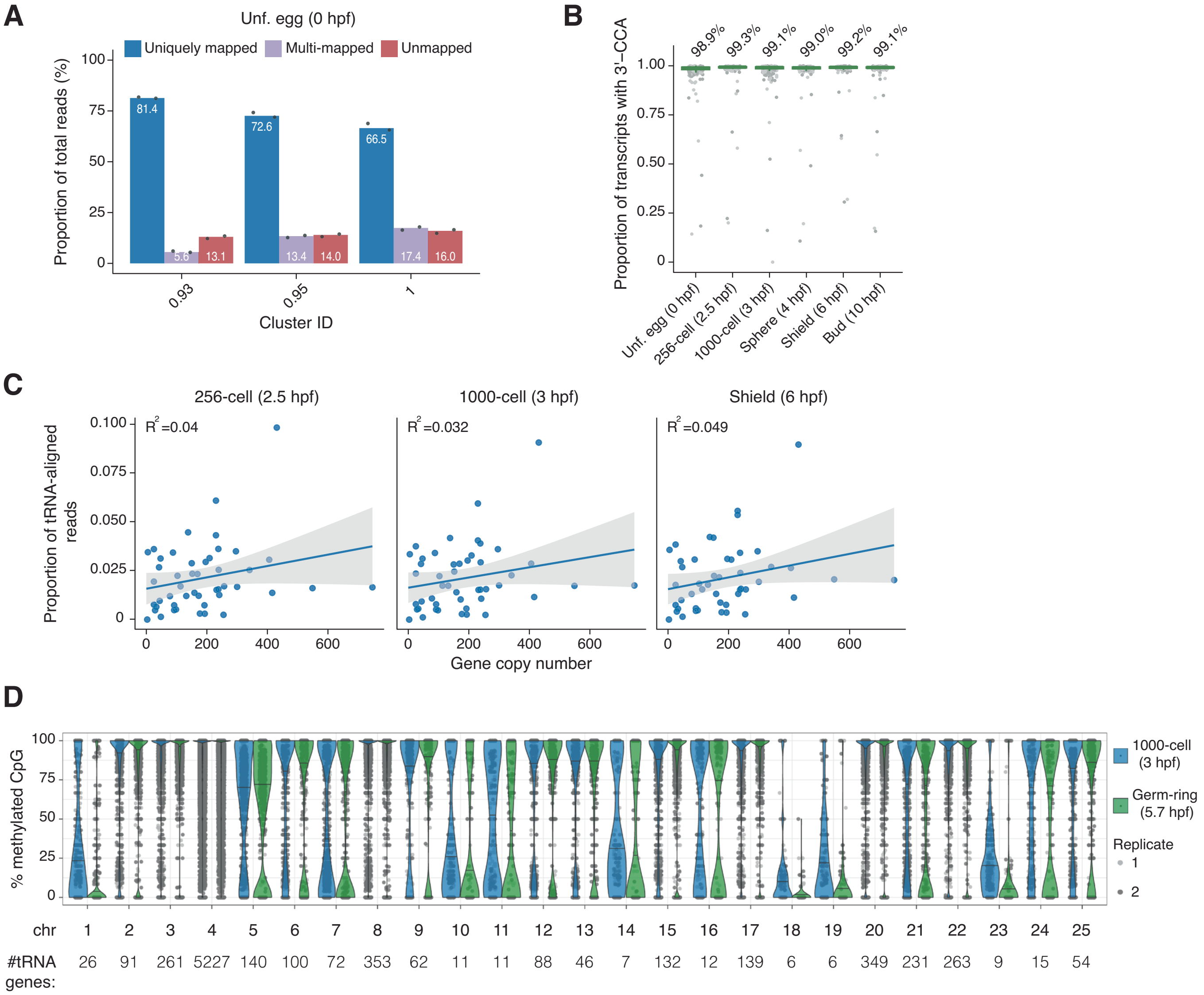

Figure EV1. Alignment rates of zebrafish tRNA sequencing libraries and correlation of tRNA levels with gene copy number.

(**A**) Alignment statistics for the unfertilized egg (0 hpf) samples ($n = 2$) for different cluster ID thresholds using the mim-tRNAseq computational pipeline (Behrens and Nedialkova, 2022). (**B**) Box plots of the proportion of tRNA transcripts containing post-transcriptional 3′-CCA additions at each of the developmental time-points determined by mim-tRNA seq ($n = 2$; central line and label: median; box limits: upper and lower quartiles; whiskers: 1.5 × interquartile range). (**C**) Correlation plots of unique tRNA gene copy number and corresponding proportion of uniquely aligned tRNA reads in single replicates ($n = 1$) for the indicated developmental time-points. Blue lines: linear regression model; shaded gray: 95% confidence interval (CI); r1 and r2: replicate 1 and 2, respectively. (**D**) Violin plots of CpG methylation proportions at tRNA genes (+125 bp upstream sequence; center line: median, $n = 2$) per standard chromosome (chr) measured by whole-genome bisulfite sequencing of 1000-cell (3 hpf) and germ ring (5.7 hpf) embryos in two biological replicates (data from Jiang et al (2013)). The number of predicted high-scoring tRNA genes (Chan and Lowe, 2016) on each chromosome in the GRCz11 genome assembly is indicated.

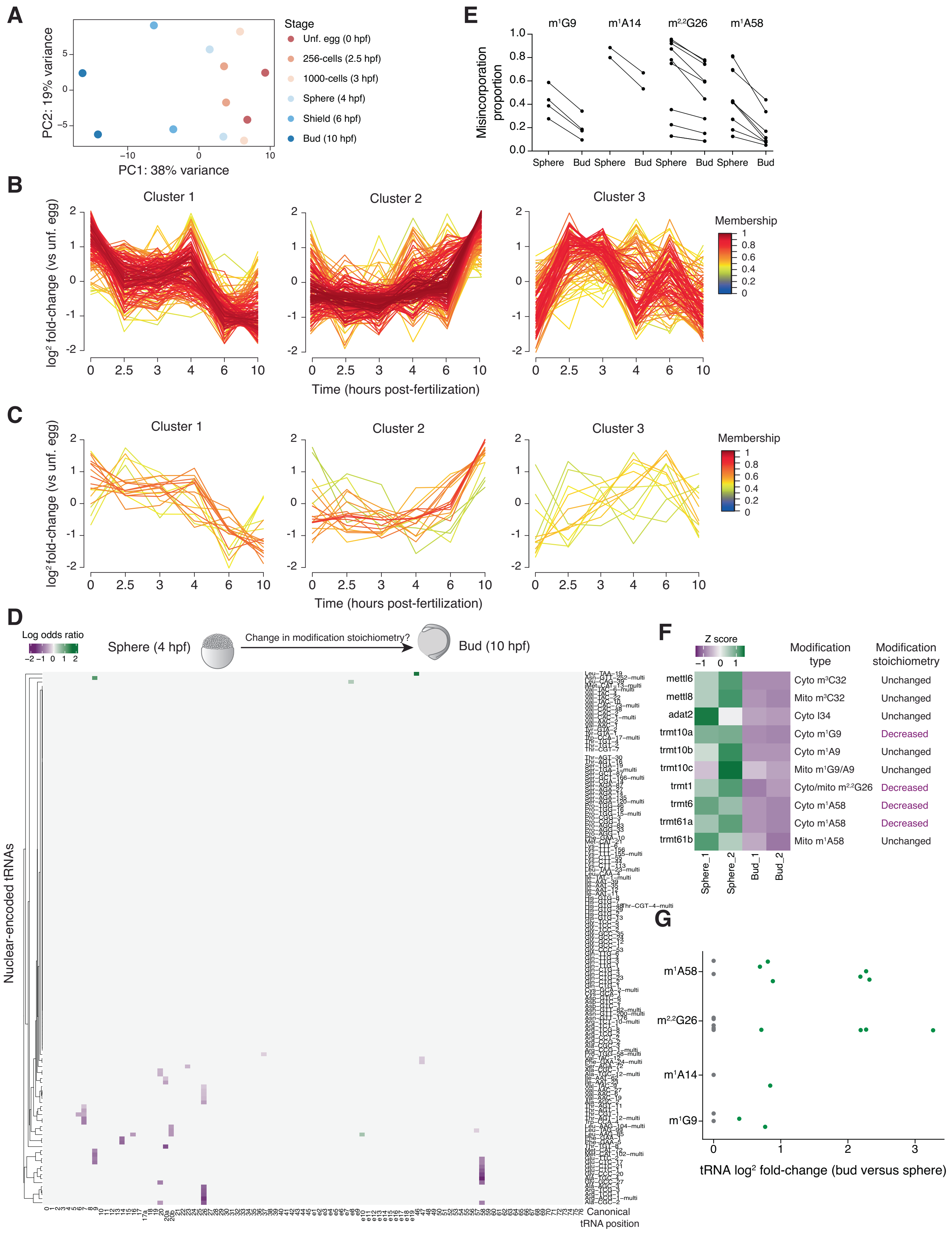
A
PC2: 19% variance
PC1: 38% variance
Stage
Unf. egg (0 hpf)
256-cells (2.5 hpf)
1000-cells (3 hpf)
Sphere (4 hpf)
Shield (6 hpf)
Bud (10 hpf)
E
m1G9
m1A14
m2,2G26
m1A58
Misincorporation proportion
Sphere
Bud
B
Cluster 1
Cluster 2
Cluster 3
log2 fold-change (vs unf. egg)
Time (hours post-fertilization)
Membership
C
Cluster 1
Cluster 2
Cluster 3
log2 fold-change (vs unf. egg)
Time (hours post-fertilization)
Membership
D
Log odds ratio
Sphere (4 hpf)
Change in modification stoichiometry?
Bud (10 hpf)
Nuclear-encoded tRNAs
Canonical tRNA position
F
Z score
Modification type
Modification stoichiometry
mettl6
Cyto m3C32
Unchanged
mettl8
Mito m3C32
Unchanged
adat2
Cyto I34
Unchanged
trmt10a
Cyto m1G9
Decreased
trmt10b
Cyto m1A9
Unchanged
trmt10c
Mito m1G9/A9
Unchanged
trmt1
Cyto/mito m2,2G26
Decreased
trmt6
Cyto m1A58
Decreased
trmt61a
Cyto m1A58
Decreased
trmt61b
Mito m1A58
Unchanged
Sphere_1
Sphere_2
Bud_1
Bud_2
G
m1A58
m2,2G26
m1A14
m1G9
tRNA log2 fold-change (bud versus sphere)

 © The Author(s)

Figure EV2. Analysis of tRNA transcripts, anticodon families, and differential modification stoichiometry during the zebrafish MZT.

(**A**) Principal Component Analysis (PCA) plot of normalized unique tRNA transcript levels for all 12 mim-tRNAseq samples. (**B**) Fuzzy c-means soft clustering analysis of expression changes during the zebrafish MZT at the level of unique tRNA transcripts and (**C**) tRNA anticodon families. tRNAs for which cluster membership value is >0.6 were considered as part of the same cluster. (**D**) Hierarchically clustered global heatmap of log odd ratios of average misincorporation ($n = 2$) in unique cytosolic tRNA transcripts in bud (10 hpf) versus sphere (4 hpf) stages. Log odd ratios were filtered for significance (Chi-square FDR-adjusted p-value ≤ 0.01) and effect size (average misincorporation log2 fold-change ≥0.5) for sites detected as modified by mim-tRNAseq. Column names show canonical tRNA position. (**E**) Scatter plot of average ($n = 2$) misincorporation proportions in sphere (4 hpf) and bud (10 hpf) for the indicated modified sites in tRNAs with significantly different log odds ratios from (**D**). (**F**) Gene expression heatmaps for selected tRNA-modifying proteins in sphere (4 hpf) and bud (10 hpf) stages ($n = 2$). Standardized Z scores were calculated from DESeq2-normalized RNA-seq counts per gene across samples. Modification type and position in nuclear-encoded (cyto) and mitochondrial (mito) tRNA is shown along with annotation of significant changes in stoichiometry inferred from misincorporation rates analysis in (**D**). (**G**) Scatterplots of log2 fold change in abundance estimated by DESeq2 for tRNA transcripts and clusters with significant changes in misincorporation frequency at the indicated modified sites in bud versus sphere (from Fig. EV2D).

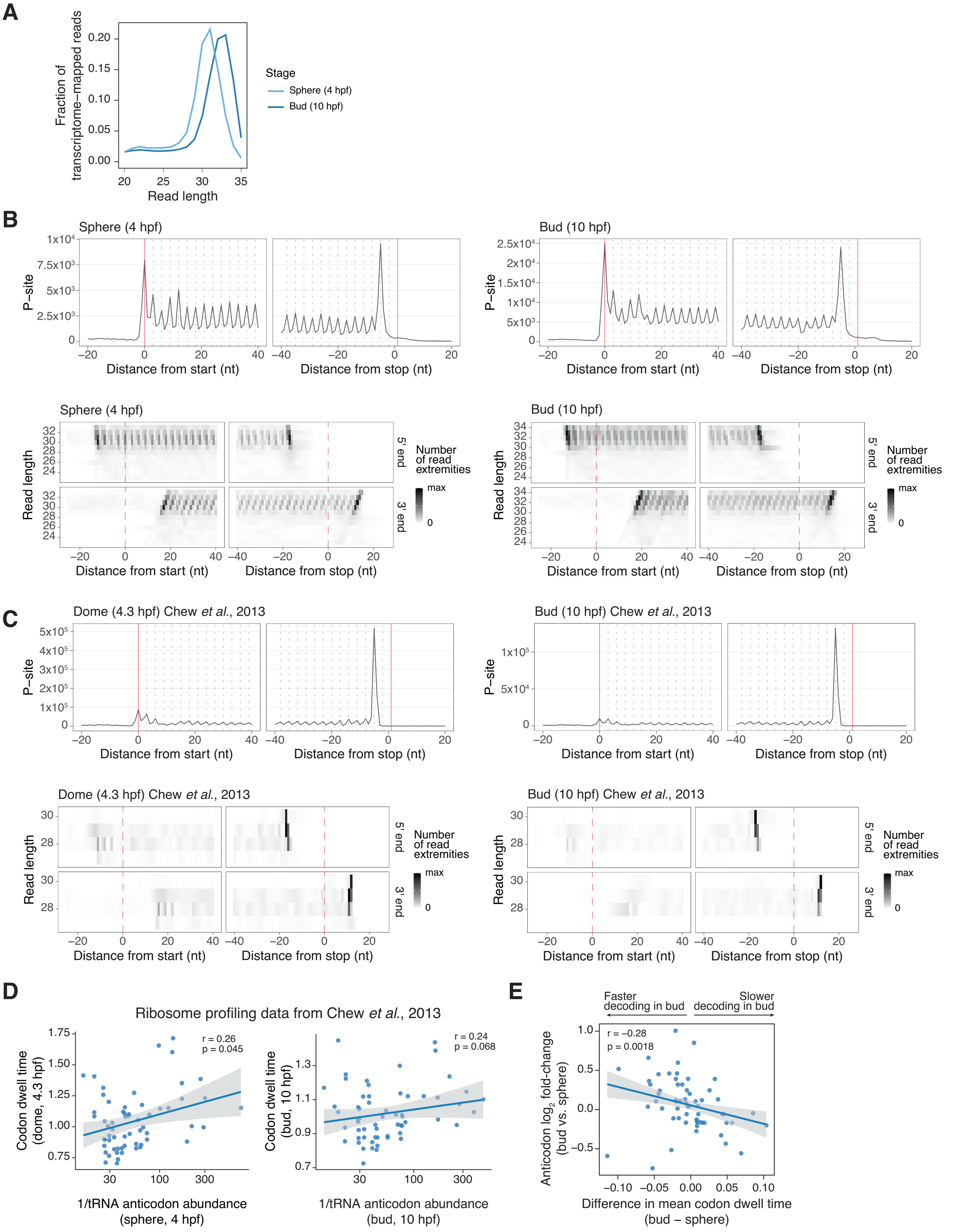
A
Fraction of transcriptome-mapped reads
Read length
Stage
Sphere (4 hpf)
Bud (10 hpf)
B
Sphere (4 hpf)
Bud (10 hpf)
P–site
Distance from start (nt)
Distance from stop (nt)
Read length
5' end
3' end
Number of read extremities
max
0
C
Dome (4.3 hpf) Chew et al., 2013
Bud (10 hpf) Chew et al., 2013
D
Ribosome profiling data from Chew et al., 2013
r = 0.26
p = 0.045
r = 0.24
p = 0.068
Codon dwell time (dome, 4.3 hpf)
Codon dwell time (bud, 10 hpf)
1/tRNA anticodon abundance (sphere, 4 hpf)
1/tRNA anticodon abundance (bud, 10 hpf)
E
Faster decoding in bud
Slower decoding in bud
r = −0.28
p = 0.0018
Anticodon log2 fold-change (bud vs. sphere)
Difference in mean codon dwell time (bud – sphere)

 © The Author(s)

Figure EV3. Relationship between tRNA levels and ribosome decoding speed at the sphere and bud stages of zebrafish embryogenesis.

(**A**) Representative read length distributions of ribosome footprints from sphere (4 hpf) and bud (10 hpf) stage samples generated by halting elongation with cycloheximide and tigecycline (**B**) Top: Meta-profiles depicting the periodicity of ribosome footprints at a transcriptome-wide scale at each stage of development based on P-site identification with riboWaltz. Bottom: Meta-gene heatmap depicting the signal associated to the 5′ and 3′ ends of the reads aligning around the start and stop codons for different ribosome footprint lengths at each stage of development. (**C**) Same as in (**B**) for publicly available ribosome profiling data by Chew et al (2013). (**D**) Correlation plots of codon-specific dwell times calculated with Scikit-ribo using ribosome profiling data from Chew et al (2013) generated by halting elongation only with cycloheximide. The abundance of cognate tRNA levels was determined by mim-tRNA seq (this study) at equivalent developmental time-points. Pearson's *r* values and their associated *p*-values are shown. (**E**) Correlation plots of log2 fold changes in tRNA anticodon abundance at the bud (10 hpf) relative to the sphere (4 hpf) stage (p-adj ≤ 0.05) and differences in mean codon dwell time between the two stages. Solid lines: linear regression model; shaded gray: 95% confidence interval (CI); Pearson's *r* value and associated *p*-value is shown.

© The Author(s)

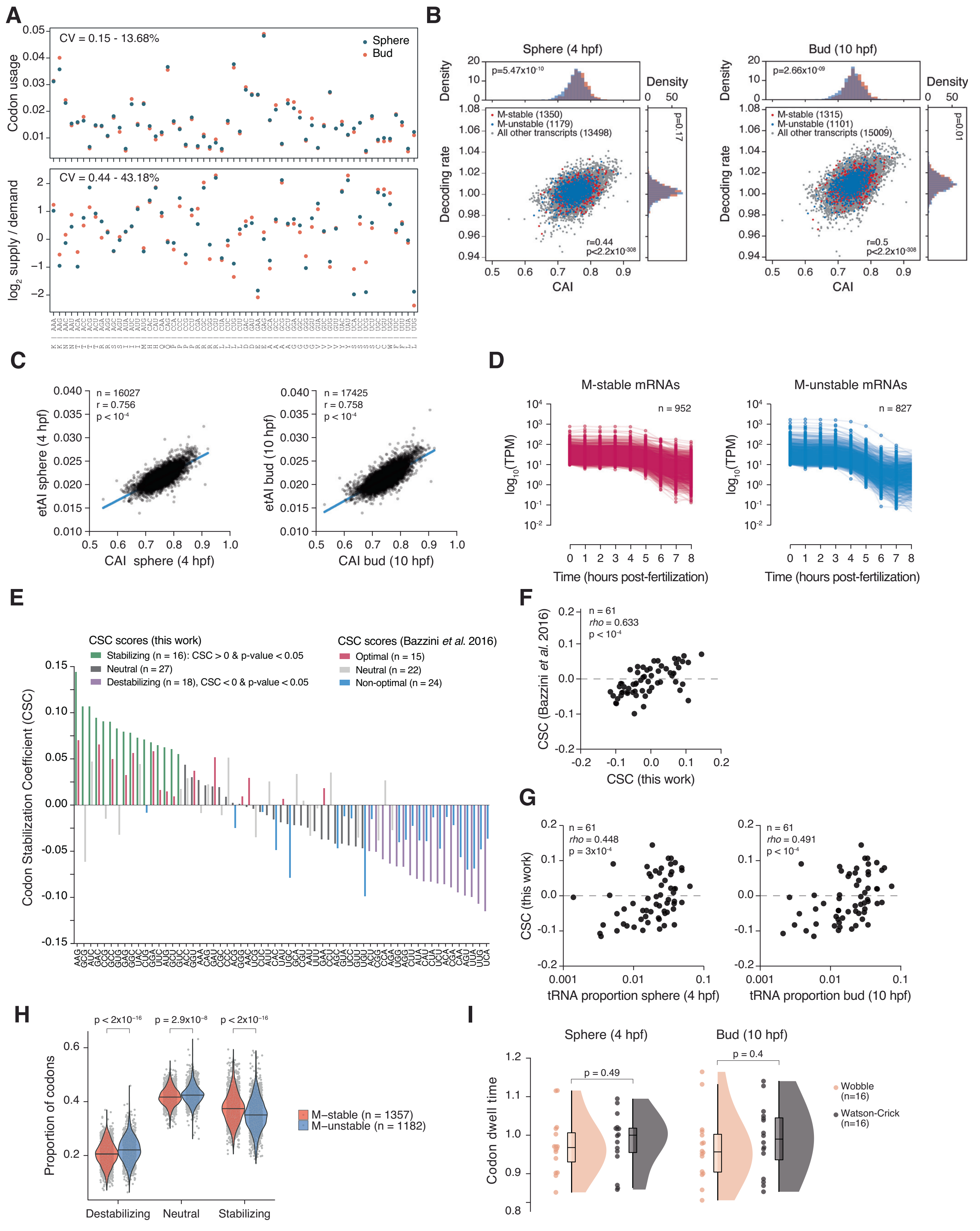
A
CV = 0.15 - 13.68%
Sphere
Bud
Codon usage
CV = 0.44 - 43.18%
log2 supply / demand
B
Sphere (4 hpf)
Bud (10 hpf)
Density
p=5.47x10-10
p=2.66x10-09
M-stable (1350)
M-unstable (1179)
All other transcripts (13498)
M-stable (1315)
M-unstable (1101)
All other transcripts (15009)
Decoding rate
CAI
r=0.44
p<2.2x10-308
r=0.5
p<2.2x10-308
p=0.17
p=0.01
C
n = 16027
r = 0.756
p < 10-4
n = 17425
r = 0.758
p < 10-4
etAI sphere (4 hpf)
etAI bud (10 hpf)
CAI sphere (4 hpf)
CAI bud (10 hpf)
D
M-stable mRNAs
M-unstable mRNAs
n = 952
n = 827
log10(TPM)
Time (hours post-fertilization)
E
CSC scores (this work)
Stabilizing (n = 16): CSC > 0 & p-value < 0.05
Neutral (n = 27)
Destabilizing (n = 18), CSC < 0 & p-value < 0.05
CSC scores (Bazzini et al. 2016)
Optimal (n = 15)
Neutral (n = 22)
Non-optimal (n = 24)
Codon Stabilization Coefficient (CSC)
F
n = 61
rho = 0.633
p < 10-4
CSC (Bazzini et al. 2016)
CSC (this work)
G
n = 61
rho = 0.448
p = 3x10-4
n = 61
rho = 0.491
p < 10-4
CSC (this work)
tRNA proportion sphere (4 hpf)
tRNA proportion bud (10 hpf)
H
p < 2x10-16
p = 2.9x10-8
p < 2x10-16
Proportion of codons
M–stable (n = 1357)
M–unstable (n = 1182)
Destabilizing
Neutral
Stabilizing
I
Sphere (4 hpf)
Bud (10 hpf)
p = 0.49
p = 0.4
Codon dwell time
Wobble (n=16)
Watson-Crick (n=16)

 © The Author(s)

Figure EV4. Relationship between mRNA codon content and codon optimality metrics (CAI and CSC) with decoding speed and tRNA levels.

(**A**) Top: Mean aggregated codon usage weighted by transcript expression (TPM) across all reference transcripts ($n = 24{,}573$) based on RNA-seq ($n = 2$, this study). Values shown correspond to the mean weighted codon usage at the sphere (4 hpf) and bud (10 hpf) stages and are represented as proportions of total codon usage values. Bottom: log2 tRNA anticodon supply to codon demand ratios per codon. Mean tRNA anticodon abundance as a proportion of all tRNA-mapped reads from mim-tRNAseq for each stage ($n = 2$) were divided by proportional mean weighted codon usages for corresponding codons. *X*-axis labels: codon sequence and corresponding amino acid in single-letter code. (**B**) Correlation plots of Codon Adaptation Index (CAI) values (calculated from the top 5% most highly expressed genes) and decoding rates at the sphere (4 hpf) and bud (10 hpf) stages. Red dots represent maternal (M)-stable mRNAs, blue dots M-unstable mRNAs and gray dots all other expressed transcripts ($n = 16{,}027$ with mean TPM > 0.5 at 4 hpf; $n = 17{,}425$ with mean TPM > 0.5 at 10 hpf in RNA-seq). Marginal density histograms for transcript decoding rates and CAI values are shown for maternal (M)-stable and M-unstable transcripts (Mann–Whitney U Test *p*-values). (**C**) Correlation plots of expression-based tRNA adaptation index (etAI) values and Codon Adaptation Index (CAI) values at each stage of development. Pearson's *r* values are shown with their significance (*p*-value). (**D**) Transcript levels of maternal (M)-stable ($n = 952$, TPM > 0.5) and M-unstable ($n = 827$, TPM > 0.5) mRNAs (classified according to Bhat et al (2023)) over time that were used for determining mRNA half-lives (this study) during zebrafish MZT. TPM time-course data from Medina-Muñoz et al (2021). (**E**) Bar plot displaying the Codon Stabilization Coefficient (CSC) value of each codon and its associated codon effect on mRNA stability during the zebrafish MZT inferred by this study and in previous work (Bazzini et al, 2016). Codons with a statistically significant ($p < 0.05$), positive Pearson correlation coefficient between occurrences of each codon in individual transcripts and their mRNA half-lives were defined as stabilizing, and codons with a statistically significant ($p < 0.05$) negative Pearson correlation coefficient were defined as destabilizing (see Dataset EV4). (**F**) Scatter plot showing the relationship between CSC values inferred by this study and in previous work (Bazzini et al, 2016). Spearman's *rho* value and associated *p*-value is shown. (**G**) Scatter plots showing the relationship between CSC values inferred by this work and corresponding anticodon tRNA proportions at each stage of development (4 and 10 hpf). Spearman's *rho* values and associated *p*-values are shown (**H**) Violin plots representing the proportion of destabilizing, neutral and stabilizing codons in maternal (M)-stable transcripts ($n = 1357$, mean TPM > 0.5 at 4 and 10 hpf) and M-unstable transcripts ($n = 1182$, mean TPM > 0.5 at 4 and 10 hpf) expressed during the MZT (Mann–Whitney U Test *p*-values). (**I**) Raincloud plots of codon dwell times calculated from ribosome footprints for wobble-decoded ($n = 16$) and Watson-Crick decoded ($n = 16$) codons at the indicated developmental stage (Mann–Whitney U Test *p*-values). Center line of box plots: median; box limits: upper and lower quartiles; whiskers: 1.5 × interquartile range. Codon dwell times were calculated from one replicate of ribosome profiling data per time-point.

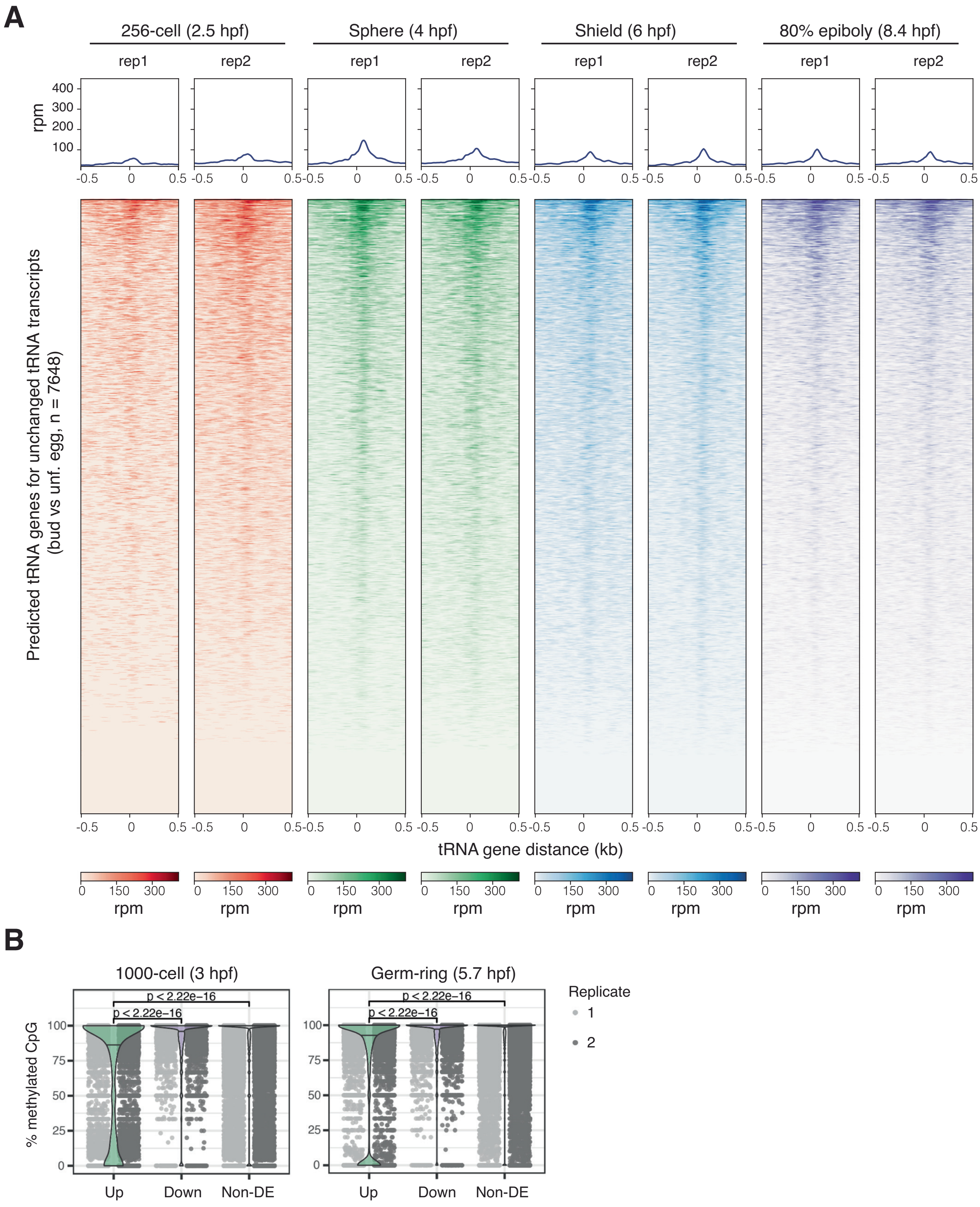
A
256-cell (2.5 hpf)
Sphere (4 hpf)
Shield (6 hpf)
80% epiboly (8.4 hpf)
rep1
rep2
rpm
400
300
200
100
-0.5
0
0.5
Predicted tRNA genes for unchanged tRNA transcripts
(bud vs unf. egg, n = 7648)
tRNA gene distance (kb)
0
150
300
rpm
B
1000-cell (3 hpf)
Germ-ring (5.7 hpf)
p < 2.22e−16
p < 2.22e−16
% methylated CpG
100
75
50
25
0
Up
Down
Non-DE
Replicate
1
2

 © The Author(s)

Figure EV5. Chromatin accessibility and methylation status of predicted tRNA genes during the zebrafish MZT.

(**A**) Global changes in chromatin accessibility at tRNA genes encoding transcripts that do not change significantly in abundance in bud (10 hpf) vs unfertilized egg ($n = 7648$) for each stage (rpm: reads per million) during the zebrafish MZT. Top: Average normalized ATAC-seq nucleosome-free region signal intensity at predicted tRNA genes. Bottom: tRNA gene start site-centered heatmaps of chromatin accessibility. Bulk ATAC-seq data from Pálfy et al, (2020). (**B**) Violin plots of CpG methylation proportions at tRNA genes (+125 bp upstream sequence; center line: median) encoding transcripts upregulated ("Up"), downregulated ("Down"), or not differentially expressed ("non-DE") during the zebrafish MZT. Data from whole-genome bisulfite sequencing of 1000-cell (3 hpf) and germ ring (5.7 hpf) embryos in two biological replicates (Jiang et al, 2013). *P*-values are from Wilcoxon tests.

© The Author(s)
